# Antiviral Strategies for Emerging Influenza Viruses in Remote Communities

**DOI:** 10.1371/journal.pone.0089651

**Published:** 2014-02-21

**Authors:** Marek Laskowski, Amy L. Greer, Seyed M. Moghadas

**Affiliations:** 1 Bartlett School of Graduate Studies, University College London, London, United Kingdom; 2 Department of Population Medicine, Ontario Veterinary College, University of Guelph, Guelph, Ontario, Canada; 3 Agent-Based Modelling Laboratory, York University, Toronto, Ontario, Canada; Northeastern University, United States of America

## Abstract

**Background:**

Due to the lack of timely access to resources for critical care, strategic use of antiviral drugs is crucial for mitigating the impact of novel influenza viruses with pandemic potential in remote and isolated communities. We sought to evaluate the effect of antiviral treatment and prophylaxis of close contacts in a Canadian remote northern community.

**Methods:**

We used an agent-based, discrete-time simulation model for disease spread in a remote community, which was developed as an *in-silico* population using population census data. Relative and cumulative age-specific attack rates, and the total number of infections in simulated model scenarios were obtained.

**Results:**

We found that early initiation of antiviral treatment is more critical for lowering attack rates in a remote setting with a low population-average age compared to an urban population. Our results show that a significant reduction in the relative, age-specific attack rates due to increasing treatment coverage does not necessarily translate to a significant reduction in the overall arrack rate. When treatment coverage varies from low to moderate, targeted prophylaxis has a very limited impact in reducing attack rates and should be offered at a low level (below 10%) to avoid excessive waste of drugs.

**Conclusions:**

In contrast to previous work, for conservative treatment coverages, our results do not provide any convincing evidence for the implementation of targeted prophylaxis. The findings suggest that public health strategies in remote communities should focus on the wider availability (higher coverage) and timely distribution of antiviral drugs for treatment of clinically ill individuals.

## Introduction

In the event of an emerging disease, of greatest concerns to public health are the geographic spread, severity, and time course of the outbreak. Determining the most effective utilization of available social, preventive, and therapeutic resources to reduce population vulnerability and mitigate disease outcomes is the main focus of the public health response. Understanding the effectiveness of control measures in population settings with distinct demographic variables and social structures can inform public health response plans for the allocation and optimal distribution of health resources prior to and during the spread of an emerging infection [1], [2].

Emerging infectious diseases are, by definition, novel in type, scope and/or distribution, and therefore pose a particular challenge for decision-makers. Decisions related to the optimal use of health resources and the implementation of community-specific intervention strategies must be made quickly and in the face of substantial uncertainty. The 2009 influenza A (H1N1) pandemic (H1N1pdm09) clearly exemplified this challenge [3]. Canada's northern remote and isolated communities were disproportionately affected by the disease and its severe outcomes, often necessitating medevac to urban areas for hospitalization and intensive care unit (ICU) admission [4], [5]. The differential rates of infection and hospitalization were particularly high in the first wave of the H1N1 pandemic in Canada [3], [6]. During the first wave, antiviral drugs were the only pharmaceutical intervention available. However, limited healthcare resources and a significant delay in the initiation of antiviral treatment post infection appear to have been barriers to realizing the full potential that antiviral drugs may have had in mitigating disease burden especially in northern communities [7]. Epidemiological data from northern Manitoba, a centrally located Canadian Province, indicate a significant delay in antiviral treatment of confirmed cases of H1N1pdm09 infection, with the mean of 3.5 days post symptoms (Figure S6 of Text S1). Clinically, in order to maximize the benefits of antiviral drugs, treatment must be initiated early (within 48 hours of symptom onset) [8].

In northern Manitoba, a significant proportion of the population self-identifies as having Aboriginal ancestry. Approximately 35% of Canadian, on reserve communities are considered semi-isolated, isolated or remote [9]. These communities range from having road access but the nearest physician services are more than 90 km away to having no road access or scheduled flights and minimal telephone and radio service [8]. Factors such as multigenerational households, challenging environmental characteristics (e.g. crowded housing, and poor indoor air quality due to tobacco use), differential prevalence of predisposing health conditions (e.g. diabetes and asthma) and other types of health disparities in these population settings (limited access to healthcare resources and high cost of nutritionally rich foods), put these communities at much higher risk for serious adverse health outcomes during a pandemic. More timely access to antiviral treatment may help to reduce the burden on the healthcare system in the event of an emerging pandemic by decreasing the number of individuals from Aboriginal communities requiring hospital and ICU admission [3].

Although not as effective or cost-effective as vaccination, antiviral use for the treatment of influenza cases is far more economical than hospitalization or intensive care. Furthermore, the time frame required for the development, production and distribution of a virus-specific vaccine using the conventional egg-based method ranges from 3 to 6 months once the virus has emerged [10], [11]. Antiviral medication will likely be the only pharmaceutical option for at least 3 months once the pandemic strain is identified and therefore the availability and strategic use of drugs is crucial for mitigating disease in remote and isolated communities, where the risk of severe outcomes appears to be significantly elevated compared to the urban populations at more southern latitudes [3], [4].

Strategically, the targeted use of antiviral drugs for the treatment of illness and/or prophylaxis of close contacts may require different policies for remote communities compared to urban populations. A “one size fits all” plan may not provide the most benefit in the case of antiviral use. Previous work has shown that demographic characteristics (e.g., age and household composition) can significantly influence the spread of disease, and therefore the impact of intervention strategies, in the population [1], [12], [13]. We sought to investigate the impact of different antiviral use strategies on the cumulative and relative age-specific attack rates (i.e., the fraction of population infected) in a synthetic population representative of a small, remote community in northern Canada. Our objectives were to: (i) assess the effect of antiviral treatment (as a single strategy) and the impact of delays in start of treatment; and (ii) evaluate the combined effect of treatment and post-exposure prophylaxis on antiviral effectiveness. For comparative evaluation, we considered the impact of similar antiviral strategies in a stylized community with the same population size, but with demographics (age, gender, employment, and household composition) shifted to resemble an urban area.

For this study, we employed an agent-based, discrete-time simulation model for the spread of a novel influenza virus in an *in-silico* population. This modelling approach allowed us to capture network patterns, and the stochasticity involved in person-to-person transmission, particularly during the early stages of the disease outbreak. A description of the model structure is provided in the *Materials and Methods* section with further details in Text S1.

### Simulation Design

We developed an *in-silico* population of individuals in a lattice-like environment consisting of homes, workplaces, school classrooms, and communal spaces that represent a remote community in northern Canada. The demographic variables (age, gender, employment, and household composition) were drawn from Statistics Canada 2006 census data [14], [15], [16]. For comparison purposes, we also created an *in-silico* environment in which the demographic variables of the remote community (but not the population size) were modified to resemble the Winnipeg health region (Figure S3–S5 in Text S1), an urban center in the province of Manitoba with a large Aboriginal population.

The agent-based simulation model is comprised of several modules: (i) a spatial module that divides the *in-silico* population into a number of workplaces, homes, school classrooms, and common spaces; (ii) a temporal module for tracking and advancing the time in simulations; (iii) an agent demographics module that maintains attributes of each agent (such as age); (iv) an agent schedule module that determines the location of each agent at any particular time; (v) a disease transmission module that governs the spread of the pathogen in the population; and (vi) a disease progression module that records and updates the change in the epidemiological status of each individual agent (Figure S1 in Text S1).

All the simulation results presented here are averaged over 1000 independent realizations, where each realization was seeded randomly with an initial infectious case. For each independent realization, agents' characteristics were drawn and assigned from distributions provided in demographic data. We set the time variable in each simulation to advance in increments of one hour. Each simulation began at the start of the pre-symptomatic infectious period for the initial infectious case, and ended when individuals were only susceptible or recovered (i.e., no exposed, pre-symptomatic, or infectious cases were encountered in the population at the current simulation time-step).

The model was implemented using a custom C++ based simulator that can take advantage of desktop and compute cluster environments. Simulations were carried out on Sharcnet (Shared Hierarchical Academic Research Computing Network), as part of the Compute Canada consortium. Following completion of simulations on Sharcnet, the resulting outputs were downloaded to the ABM-Lab 64 core SMP machine [17], and analyzed using Perl and MATLAB. Further details of the model simulator are provided in the *Materials and Methods* section.

## Results

Table 1 summarizes the scenarios simulated and discussed here for an estimated reproduction number *R*_0_ = 2.2 for the RC population. To capture the effect of changes in reproduction number, we also calibrated and simulated the model for *R*_0_ = 1.6 and 2.8. (Figures S13–S20 of Text S1).

**Table 1:** Simulation scenarios presented for treatment and prophylaxis strategies, when prophylaxis was implemented for 3 weeks.

Simulation scenarios	Simulation outcomes	Demographic scenario	*R*_0_	Delay in start of treatment		
				1 day	2 days	3 days
Treatment, no prophylaxis	Age-specific cumulative AR*	RC	2.2	Fig1a	Fig1b	Fig1c
		SD	1.4	Fig1d	Fig1e	Fig1f
	Age-specific relative AR	RC	2.2	Fig2a	Fig2b	Fig2c
		SD	1.4	Fig2d	Fig2e	Fig2f
Treatment and prophylaxis	Overall AR	RC	2.2	Fig3a	Fig3b	Fig3c
		SD	1.4	Fig4a	Fig4b	Fig4c
	Wasteful use of prophylaxis	RC	2.2	Fig3d	Fig3e	Fig3f
		SD	1.4	Fig4d	Fig4e	Fig4f
Treatment and prophylaxis	Effective use of drugs	RC	2.2	Fig6a	Fig6b	Fig6c
		SD	1.4	Fig6d	Fig6e	Fig6f

Simulation outcomes for *R*_0_ = 1.6 and 2.8 are reported in Figures S13–S20 of Text S1.

^*^ AR: attack rate.

### Treatment and prophylaxis strategies

We first considered antiviral strategies for treatment of identified infectious cases with the circulating influenza strain. We then implemented an additional post-exposure prophylaxis strategy in which close contacts of identified infectious cases were offered prophylaxis. A newly identified infectious case is defined as an agent that started receiving treatment, but not as a result of contact-tracing identification (i.e., seeking treatment or self-identify themselves). The treatment and prophylaxis coverages were varied from 0 to 100% (in increments of 2%). Treatment coverage refers to the fraction of infectious individuals receiving treatment, and prophylaxis coverage refers to the fraction of close contacts (of treated cases) that receives prophylaxis. To ensure that prophylaxis is offered early on during the latent or pre-symptomatic period, we considered prophylaxis for only close contacts occurring in the 24 hours prior to the onset of treatment for an infectious case. We assumed that treatment continued for the entire infectious period of an infectious case, which was sampled from a log-normal distribution for every infected individual (see Text S1). Close contacts who developed disease while receiving prophylaxis continued antiviral therapy with treatment at the increased dosage for the entire sampled infectious period.

We allowed re-prophylaxis of individuals who completed a course of prophylaxis without developing disease, and were subsequently identified as a close contact of another identified infectious case. Prophylaxis was offered if the contact was identified at least 24 hours after the completion of a previous course of prophylaxis. For antiviral strategies, we considered three scenarios for the treatment of identified infectious cases, corresponding to an average delay of 1, 2, or 3 days for the initiation of treatment after the onset of symptoms. Scenarios with longer delays between symptom onset and the start of treatment are closer to the average delay observed in H1N1pdm09 epidemiological data reported for northern Manitoba, Canada where many remote and isolated communities are found (see Text S1). In each strategy, we implemented scenarios where post-exposure prophylaxis was offered in the community for three weeks and five weeks after the first infectious case was identified for treatment. While a short-term period of targeted prophylaxis is generally feasible, long-term prophylaxis poses significant challenges once the healthcare system has been overwhelmed. For short-term scenarios, no prophylaxis was offered beyond the end of the term (in both three and five weeks scenarios), but those who started a course of prophylaxis towards the end of the program completed their antiviral regimen. Here, we describe the results for an antiviral strategy involving a three-week prophylaxis use of drugs. The results for a 5-week prophylaxis use are presented in Figures S7–S10 of Text S1.

### Age-specific attack rates

**Figure 1:**
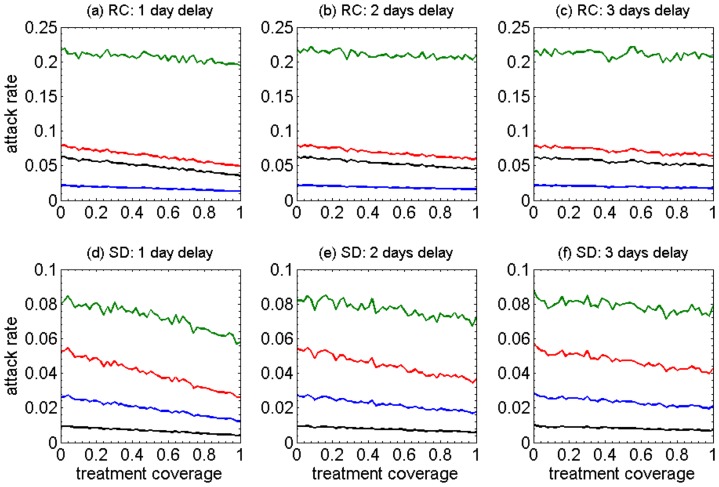
The impact of different coverages (0–100%) of antiviral treatment on the age-specific cumulative attack rates under different assumptions for the mean time between symptoms onset and the start of treatment (1–3 day delay). Panels a–c (RC) show results from the model representing the remote and isolated Canadian Aboriginal community, and panels d–f (SD) show results from the model representing a community with an urban-like shifted demographic structure. Green: School-aged children (6–18); red: adults (19–49); blue: older adults (50+); black: pre-school (0–5).

The simulation outputs were analyzed for four main age groups in the population: pre-school children (0 to 5 years of age); school-aged children (6 to 18 years of age); adults (19 to 49 years of age); and older adults (50+ years of age). We calibrated the model to ascertain the probability of transmission, from one infectious agent to one susceptible contact, using the estimated average 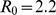 for northern Manitoba [18], [19]. With antiviral treatment only and in the absence of prophylaxis, the variations in age-specific, cumulative attack rates with different treatment coverage are shown in Figure 1a–c. School-aged children have considerably higher attack rates in all scenarios regardless of the delay in the start of treatment following the onset of symptoms (green curves), and regardless of the treatment coverage. Also, the oldest age group (50+ years of age) has the lowest attack rates in all scenarios (blue curves). We observed that increasing the treatment coverage has a marginal effect on reducing cumulative attack rates for the children and older adults, and the delay in start of treatment has virtually no impact on the magnitude of this reduction. However, increasing the treatment coverage can have a relatively modest effect on reducing attack rates in other age groups (red and black curves). Longer delays in the start of treatment would decrease this effect particularly for high coverages of treatment.

For comparison purposes, we shifted (modified) the demographic variables of the remote community (RC) to resemble those of the Winnipeg health region, the largest urban centre in the province of Manitoba, which has a significant urban Aboriginal population. When averaging independent realizations in the absence of treatment, we found 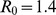 for the model with shifted demographics (SD). Simulation results of cumulative, age-specific attack rates for the SD are shown in Figure 1d–f. Compared to the scenarios for the original RC demographics presented in Figure 1a–c, we observed several important differences. Most conspicuous is the results that the lowest attack rates are associated with the pre-school children (below 2%), while the attack rates for the group aged 50 years and older remain in the same range 2%–4%. The attack rates for school-aged children (green curves) in all scenarios of SD are significantly lower (below 10%) than those of the original RC demographics (above 20%). Overall, the reduction in age-specific attack rates is comparable in the corresponding scenarios for treatment delay between SD and the original RC demographics.

**Figure 2:**
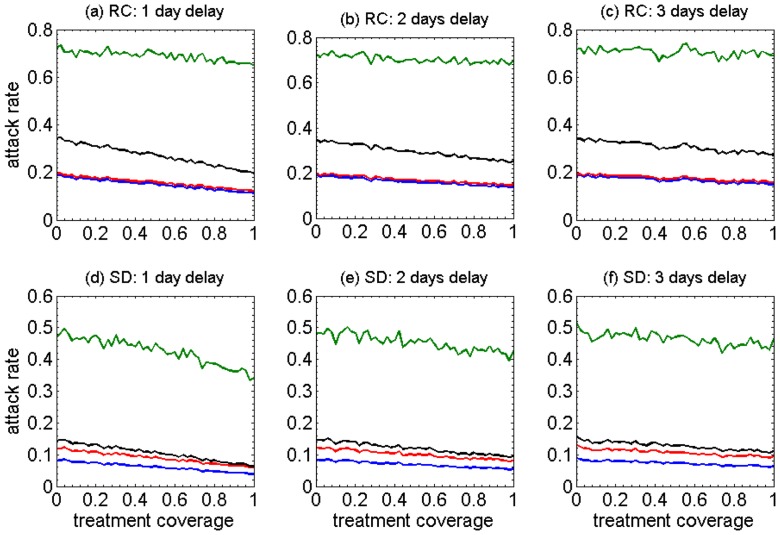
The impact of different coverages (0–100%) of antiviral treatment on the age-specific relative attack rates under different assumptions for the mean time between symptoms onset and the start of treatment (1–3 day delay). Panels a–c (RC) show results from the model representing the remote and isolated Canadian Aboriginal community, and panels d–f (SD) show results from the model representing a community with an urban-like shifted demographic structure. Green: School-aged children (6–18); red: adults (19–49); blue: older adults (50+); black: pre-school (0–5).

We also analyzed the simulation outputs for the relative attack rates (the fraction of infected individuals in each age group) for all the scenarios discussed above. The results of age-specific, relative attack rates are shown in Figure 2a–f. Interestingly, the highest attack rates occur in the school-aged children within the ranges 60%–80% and 30%–50% in the RC and SD demographics, respectively. Attack rates for pre-school children are below school-aged children, and lie within the ranges 20%–40% and 8%–20% in the RC and SD demographics, respectively; but remain above those of adults and 50+ age groups in all scenarios. While equivalent in the original demographics of RC, attack rates of adults remain slightly above the group aged 50 years and older in SD scenarios. Overall, increasing treatment coverage decreases the relative attack rates in each age group, but the delay in the start of treatment reduces this effect.

Overall, in the model with RC demographics, increasing antiviral treatment coverage of infectious cases can have a positive impact on decreasing the attack rates in all age groups. The effect is most easily observed when comparing relative attack rates rather than cumulative attack rates. However, if treatment is not administered within 24 hours after symptom onset, the observed effect is very minimal. The ordering of cumulative attack rates differs between the RC and SD demographics. This observation is a function of the different age structure of the populations with the RC population having many fewer individuals in the oldest age category (50+).

### Combined effect of treatment and prophylaxis

**Figure 3:**
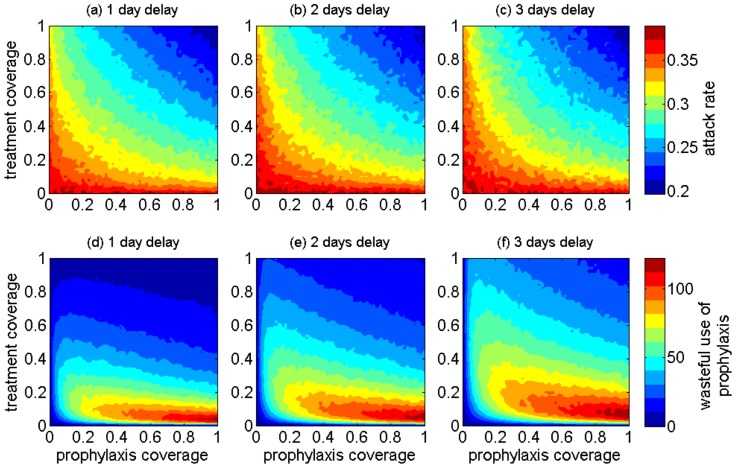
The projected effect of combining antiviral treatment of infectious cases with prophylaxis of close contacts on population attack rates (a–c) and wasteful use of drugs (d–f) in the RC demographics when antiviral treatment delays (for infectious cases) range from 1 day to 3 days post symptom onset.

**Figure 4:**
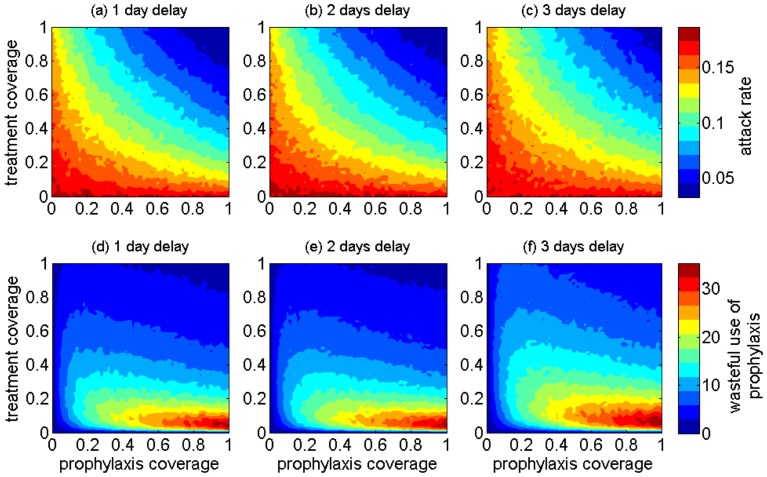
The projected effect of combining antiviral treatment of infectious cases with prophylaxis of their close contacts on population attack rates (a–c) and wasteful use of drugs (d–f) in the SD demographics when antiviral treatment delays range from 1 day to 3 days post symptom onset.

We ran simulations for both the RC and SD demographics when the treatment of identified infectious cases is augmented with prophylaxis of their close contacts (defined as individuals having direct contact with the case in the 24 hours prior to case identification). Simulation results of the cumulative attack rates and “wasteful use” of prophylaxis are presented in Figure 3a–f for the RC demographics, and Figure 4a–f for SD demographics. In our model, the “wasteful use” of prophylaxis refers to the use of antiviral drugs for those who were identified as close contacts of infectious cases under treatment, but who were previously infected and not diagnosed. In this case, administering antiviral treatment to these individuals is considered “waste”, because they will not develop disease as a result of their close contact. This is due to the fact that in our model, we assumed that the immunity generated during primary infection prevents re-infection by the same influenza strain.

For low treatment coverage (below 10%), increasing prophylaxis coverage of close contacts has little impact on reducing attack rates (Figure 3a–c). However, the effect of prophylaxis becomes more pronounced as the treatment coverage increases above 40%. The greatest waste of prophylaxis corresponds to the range 5%–20% treatment coverage in the scenario of 1-day delay for start of treatment. For longer delays, the range of prophylaxis waste expands to higher treatment coverages (Figure 3d–f). The overall waste of prophylaxis is significantly higher in the model with the original RC demographics compared to SD, and could be as much as 3 times higher in the range 10%–40% treatment coverage, which is considered a plausible range for public health to successfully implement an antiviral treatment policy [20], [21]. This relatively low treatment coverage may result from several factors, including diagnosis uncertainties for influenza cases, treatment guidelines for use of antiviral drugs, familiarity with antiviral agents, access to drug stockpiles, or knowledge of the potential severity and outcomes of infection. Our simulations suggest that, when the treatment coverage is relatively low (below 40%), prophylaxis of close contacts should be targeted at a low coverage (below 10%) to avoid excessive waste.

### Attack rates at different community places

**Figure 5:**
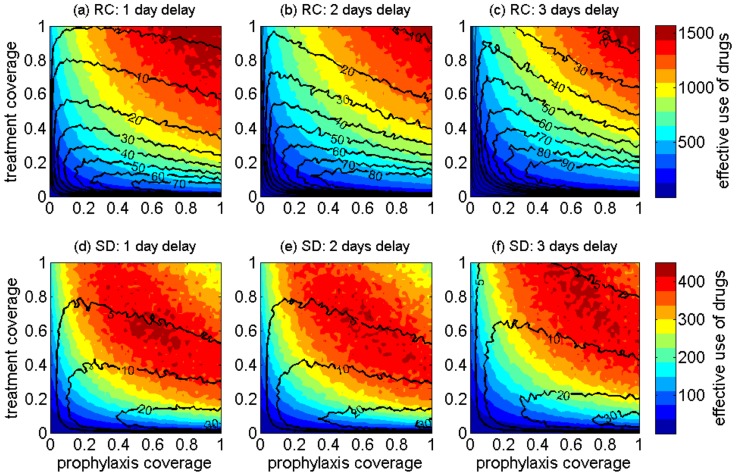
Box plots for the projected attack rates in households, workplaces, schools, and other places for RC and SD demographics with 1-week prophylaxis strategy. Red circles are outliers for boxplots between the first and third quartiles.

We analyzed the simulation results for attack rates in different locations of the modeled community: households, workplaces, schools, and other places. From multiple scenarios, we chose to present the results for a 1-day delay in start of treatment combined with the scenario of 3 weeks prophylaxis for both RC and SD demographics. Figure 5 shows boxplots for these simulations, indicating that in contrast to the SD demographics, attack rates among households could be significantly higher than those for workplaces (possibly due to more crowded households in the RC compared to SD demographics). In all scenarios, the highest attack rates correspond to schools for both RC and SD demographics. Prophylaxis can have a modest impact on reducing household attack rates in the RC demographics, but this effect is relatively marginal on attack rates associated with other locations.

### Drug-use

**Figure 6:**
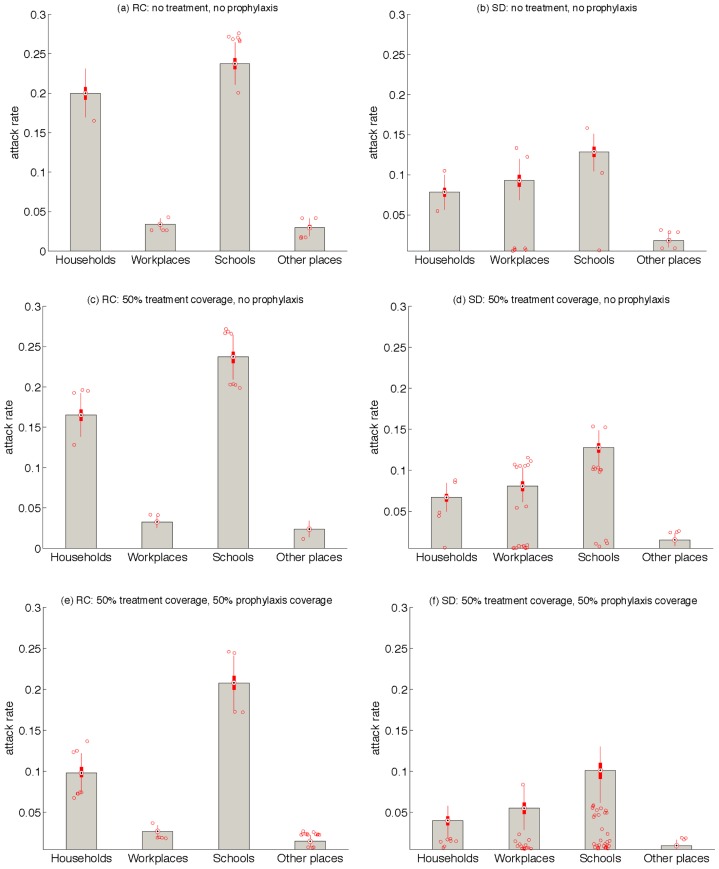
The projected effective use of antiviral drugs when combining antiviral treatment of infectious cases (y-axis) with prophylaxis of close contacts (x-axis) in the remote community model (RC) (a–c) and the shifted demographics model (SD) (d–f). Antiviral treatment delays range from 1(counted in the number of courses given) for dispensing drugs as prophylaxis.

For antiviral strategies combining treatment of infectious cases with prophylaxis of close contacts, we analyzed the simulation outputs for the effective use of drugs (defined as the total drug usage excluding wasteful use of prophylaxis). The number of effective courses of drugs used is presented as heat-maps in Figure 6a–f for the RC and SD demographics models. Contour curves in Figure 6 correspond to the wasteful use of prophylaxis. Heat-maps illustrate that the effective use of drug depends critically on the coverages for treatment and prophylaxis. For an antiviral strategy with a 1-day delay in the start of treatment after the onset of symptoms, the largest drug use occurs in the high range of treatment and prophylaxis (Figure 6a). This range is lower (in the middle) for the same antiviral strategy in the SD population (Figure 6d). In the SD demographics, high coverages of treatment and prophylaxis are associated with lower use of drugs compared to the middle range. For the RC demographics, as delay in the start of treatment increases to 3 days, a transition in the range for effective use of drugs moves toward higher coverages of treatment and prophylaxis. These results suggest that, in the case of limited drug supply, early treatment with high coverages of treatment and prophylaxis will provide maximum benefits both in terms of drug usage and the reduction of attack rates.

## Discussion

Our study, for the first time, provides a comparative evaluation of antiviral strategies for mitigating the effect of a novel influenza virus in populations with distinct demographic characteristics, representative of a remote community and an urban community in Canada. We compared the outcomes of treatment-only and combined treatment-prophylaxis strategies in terms of cumulative and relative attack rates, drug usage, and wasteful use of prophylaxis in four different age groups in the population. Qualitatively, we observed several similarities and differences between the observed outcomes. First is the observation that in crowded settings with low average age (e.g. RC populations), the highest attack rate is associated with school-aged children. Delay in treatment interventions following the onset of symptoms has a large impact on the effectiveness of antiviral drugs in reducing the overall and age-specific attack rates (Figure 1). These observations can be placed in the context of remote communities of primarily Aboriginal ethnicity. In these communities, multigenerational households and strong community social ties can lead to frequent close contacts allowing influenza to spread throughout the community relatively easily. When comparing the relative attack rates for each age-group, we observed similar patterns for both remote and urban-like demographics, with the highest and lowest attack rates in school-aged children and individuals aged 50 years and older, respectively. These findings suggest that a significant reduction in relative attack rates of a specific age group may not necessarily contribute to a large reduction in the cumulative attack rates.

When treatment is implemented alongside prophylaxis of close contacts, we observed similar patterns for the overall attack rate in both models for original (RC) and shifted (SD) demographics. Importantly, the wasteful use of drugs for prophylaxis corresponds to levels of antiviral treatment that are generally most plausible in public health responses (between 10% and 40%) [20], [21]. Our simulations suggest that, in both demographic scenarios, when treatment coverage varies from low to moderate, targeted prophylaxis should be offered at only low levels (below 10% in our simulations) to avoid excessive waste of drugs (Figures S11 and S12 of Text S1). Within an antiviral strategy for post-exposure prophylaxis, early treatment of infectious cases is more crucial to the effectiveness and effective use of drugs.

Maintaining a significant public health response that focuses on following up close contacts of infectious cases for the provision of antiviral prophylaxis for a time period in excess of 3 weeks described here (e.g., 5 weeks) has very little impact on overall attack rates, and results in a considerably more drug wastage (*see* Text S1). Furthermore, such an extended period of prophylaxis contributes to a significant workload to an already overburdened health system, particularly during the peak incidence of infection. We observed qualitatively similar behaviours by simulating our models for different reproduction numbers (see Text S1).

When treatment is maintained at conservative levels, our findings do not provide any convincing evidence for the implementation of community-wide prophylaxis of close contacts. These findings also suggest that the early initiation of antiviral treatment is more critical for lowering attack rates in a crowded setting with a low average age (such as a remote community with demographic characteristics simulated here) compared to an urban-like population. Furthermore, a longer delay in the start of treatment significantly diminishes the effect of increasing treatment coverage in reducing the overall attack rate (Figures 3–4).

There is an emerging body of evidence suggesting that there is a strong correlation between the speed with which antiviral treatment is initiated following the onset of symptoms and the degree of disease severity in critically ill patients of influenza [3], [4]. The experience of H1N1pdm09 in Canada's northern Aboriginal communities provides compelling evidence for severe disease outcomes, often necessitating hospitalization and ICU admission [3], [4], [6]. Analysis of the age-distribution of the H1N1pdm09 cases in the province of Manitoba, Canada, indicates significantly higher rates of infection and hospitalization amongst First Nations compared to non-First Nations populations. These rates were as much as 12 times (for infection) and 22 times (for hospitalization) higher in First Nations young children, aged 0–4, compared to the same age group in non-First Nations populations [6]. Continued improvements to the health infrastructure in northern communities during the inter-pandemic period and focused pandemic planning will permit northern healthcare providers to more effectively manage the surging demand for healthcare and specifically antivirals during the peak of pandemic outbreaks [3].

### Model limitations

Any model, regardless of the level of detail included in its structure, is subject to limitations. While several limitations are related to the lack of specific data and measurements, others arise from the assumptions. For example, the mitigation effects of antiviral drug use on disease spread can be modeled as a reduction of disease transmissibility from the start of treatment but no reduction in infectious period, or a reduction in infectious period but no reduction in transmissibility, or a combination thereof. In a practical sense, the overall effect of treatment should match the reduction in secondary attack rates observed in household studies [22], [23]. Since reduction in transmissibility over the entire infectious period can capture the effectiveness of antivirals, we chose the first method. We also did not consider antiviral use in the context of other public health interventions; however, a variety of interventions (such as vaccination if available, quarantine/isolation, or social distancing including school closure) may take place simultaneously during the course of an outbreak.

Our model does not explicitly include asymptomatic infection; however, a distribution of transmission probabilities in infectious individuals would address the variability in infectiousness seen in undiagnosed infectious cases, of which the asymptomatic infections are a subset. Incorporating a separate class for asymptomatic individuals in the model can be managed computationally, but we have steered clear of this additional class in the model because very little is known about asymptomatic cases, and so the challenge of finding appropriate data would be significant. Yet, we understand that antiviral wastage may in fact exceed what we have described here, due to the possibility of asymptomatic infections. We only consider waste as a result of giving prophylaxis to “recovered” individuals that were never identified as cases and therefore recovered naturally from their infection and would be immune against re-infection with the same strain of influenza. We did not consider antiviral wastage that might occur as a result of the co-circulation of other pathogens that cause acute respiratory disease [24]. Furthermore, our model does not take into account the evolution of drug resistance. In the context of transmissible drug-resistance, prophylaxis would only enhance the spread of resistant strains [25], [26]. This is facilitated by a reduction of susceptibility to the sensitive infection and an increase in vulnerability of susceptible individuals who are receiving prophylaxis to resistant infection.

Other limitations related to the lack of specific data include our basic models for the time-use of individuals during their regularly scheduled activities, and the level of strain-related pre-existing immunity in the population. The type of community modeled is considered to be remote and isolated, and therefore interactions with other communities are not modelled. Finally, the model outcomes are quantitatively subject to uncertainty in disease parameters. We performed simulations based on recent parameter estimates of H1N1pdm09 data and previous estimates of antiviral effectiveness. However, the combination of treatment and prophylaxis is not adequately discussed in the literature, particularly for remote and isolated communities. While quantitative results of our simulations may change with variations in model parameters, or the choice of modelling approach, we expect our results to remain qualitatively robust despite the above limitations.

## Materials and Methods

We restructured and calibrated a previously validated agent-based modelling framework for the spread of influenza infection in a remote and isolated community in northern Canada, referred to as RC throughout this paper [1]. We included antiviral treatment of identified infectious cases, and post-exposure prophylaxis of close contacts as control measures in the absence of pre-existing immunity or vaccination.

### Modelling framework

**Figure 7:**
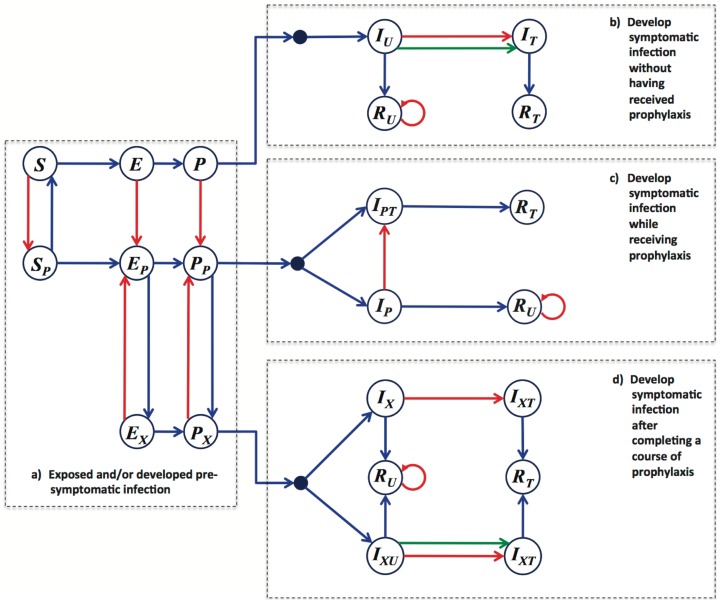
Compartmentalization of the model structure with four main blocks: (a) prior to becoming symptomatically infectious; (b) develop symptomatic infection without having received prophylaxis; (c) develop symptomatic infection while receiving prophylaxis; (d) develop symptomatic infection after completing a course of prophylaxis. Subscripts have the following meaning: (*P*) the agent is currently undergoing a course of prophylaxis; (*X*) the agent is (was) receiving prophylaxis during the pre-symptomatic or exposed period; (*U*) the agent is unidentified and therefore not treated (when Infectious, following a delay, the agent may seek treatment with some probability); (*T*) the agent is under treatment (or received treatment), and is therefore known to have had the disease. Blue arrows indicate disease progression, which is influenced by the passage of time and some particular disease parameters. In the case of the S-to-E transition, transmission occurs between co-located susceptible and infectious individuals in either pre-symptomatic or symptomatic stage. Green arrows indicate self-identification following a delay in seeking treatment by a symptomatically infectious agent. Red arrows are socially mediated transitions resulting from the identification (i.e., contact-tracing) of the agent as having been in contact with a new infectious case within the last 24 hours.

To capture the movement and interactions of individuals (i.e., agents), we developed an *in-silico* environment as a lattice-scaled representation of the community. We considered a Markov Chain compartmental structure for disease spread, and assumed that at any particular time, each agent belongs to one of the compartments in the model depending on the particular epidemiological history of that agent. The basic framework includes the compartments represented in Figure 7, which correspond to susceptible, exposed, pre-symptomatic, symptomatic, and recovered classes of agents. To include the effect of antiviral treatment, we further subdivided these compartments by using subscripts. The transition between model compartments was a function of time and other model variables. The type of transition is indicated by the color of the arrows in Figure 7. A description of model variables and ranges of parameter values are provided in Table S1 and S2 of Text S1.

### Population study

We assume that the remote community (RC) has no road access to any urban center and air travel is the only possible conduit. The demographic data (i.e. age, gender, household size, and employment characteristics) for RC were based on Statistics Canada census data [27]–[29] that are rounded to the nearest five individuals. The population size of each age group, and the distribution of individuals per household for the original RC demographics are given in Figures S3–S5 of Text S1. For shifting the demographic variables to those of an urban centre (the Winnipeg health region), we modified the age distribution, household size, and employment characteristics using reported census data, while keeping the same population size of RC (see Text S1).

### Model parameterization

For each pair of co-located infectious and susceptible individuals, the probability of transmission is independent and given by
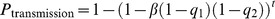
Where 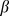 is the transmission rate; 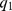
is the reduction in transmissibility (viral shedding) of an infectious individual due to antiviral treatment and/or prophylaxis; 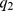 is the protective effect of prophylaxis offered to a susceptible individual; and 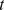 is the amount of time spent in potentially infectious contacts. We assumed that infected individuals during pre-symptomatic stage are (on average) 50% less infectious than during symptomatic phase (see Table S2 of Text S1).

In the absence of any antiviral interventions, the rate of disease transmission, 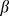, was iteratively modified through a series of trials in order to calibrate the model to an initial reproduction number, 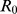, within the estimated ranges for H1N1pdm09 in northern communities [17]. In our calibration, 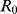 was calculated by averaging the number of secondary infections generated by the initial infectious case in each independent simulation. Each trial used 10,000 randomly initialized simulation runs. Averaged calibration trials determined the rate 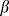, with an average 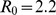 for the original RC demographics. The corresponding 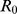 for (urban-like) shifted demographics was found to be 1.4. For calculation of relative and cumulative attack rates, we averaged the total number of infections in different age groups and overall (throughout the entire course of outbreak) for all simulation trials. This averaging excluded simulations in which no secondary infections were generated by the initial infectious case.

We included the overall effect of antiviral treatment in the reduction of disease transmissibility following the initiation of treatment. We assumed that treatment reduces the infectiousness by 60% if the infectious person was not receiving prophylaxis at the time of exposure [30]. Prophylaxis was assumed to reduce susceptibility to infection by 30% and transmissibility (if infected) by 60% [29]. Those who developed symptomatic infection while receiving prophylaxis and continued with treatment were assumed to have an additional 60% reduction in infectiousness [29], [31], with an overall 84% reduction of infectiousness (given by 1–(0.4×0.4)  = 0.84). We assumed a probability of 0.65 that individuals receiving prophylaxis will have significantly milder symptomatic infection (if they developed illness) [30], [31], and therefore will not seek treatment. A course of prophylaxis in the model simulations lasted for 7 days. Previous work [32] considered a longer duration of prophylaxis (about 10 days); however, considering possible compliance issues, we assumed a shorter prophylaxis period. Individuals that were previously receiving prophylaxis were not eligible to receive a subsequent course of prophylaxis if identified as a close contact within 24 hours of completing the previous course. Symptomatically infectious individuals who are identified as a close contact were offered antiviral treatment, unless they were symptomatically infectious for more than 48 hours prior to identification. A course of treatment for each individual spanned for the entire duration of infectiousness.

The exposed period was drawn from a uniform distribution with a minimum of 1 day and a maximum of 2 days [33]. The pre-symptomatic period for each infected individual was drawn from a log-normal distribution with the scale parameter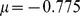 days, and shape parameter 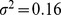 days, giving an average of 0.5 days [34]. The duration of symptomatic infection was sampled from a log-normal distribution (Figure S2 of Text S1), with the scale parameter 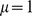 day, and the shape parameter 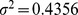 days, which has a mean of 3.38 days [35]. In our model, we used 1000 independent realizations to sample the resulting generation times in the absence of any interventions. Averaging the time interval from the start of pre-symptomatic period of the initial infectious case to the start of pre-symptomatic period of the earliest secondary case in these realizations resulted in 69.3 hours, giving a generation time of approximately 2.9 days (95% confidence interval: 25–131.3 hours), which lies within the estimated range for H1N1pdm09 [36]–[38]. Again, without interventions, we calculated the generation time throughout the outbreak [39], [40], using the same method applied to infectious cases beyond the initial case. We ran 1000 independent realizations, and averaged the individual generation times for each epidemic episode to determine the generation time distribution. We estimated the mean generation of 71.9 hours (approximately 3 days) with the 95% confidence interval 24–133 hours. These simulations provided an average doubling time of 34 hours (1.4 days) for the time interval between the introduction of the first infectious case to the time of exposure of the earliest secondary case. The delay in seeking treatment after the onset of symptoms was sampled from a uniform distribution with a minimum of 0.5 days, and a mean of 1, 2, or 3 days, corresponding to the simulated scenarios.

## Supporting Information

Text S1 **Supplementary Information.** Details of model structure, parameter values, and further simulation scenarios.
(PDF)
