# Supplementary material for: Antiviral Strategies for Emerging Influenza Viruses in Remote Communities
Source: PLoS One. 2014 Feb 21;9(2):e89651. doi: 10.1371/journal.pone.0089651 (PMC3931825; doi:10.1371/journal.pone.0089651)
Supplement: Text S1 — Supplementary Information. Details of model structure, parameter values, and further simulation scenarios. (PDF) [file pone.0089651.s001.pdf]

Supplementary Information

Antiviral Strategies for Emerging Influenza Viruses in Remote Communities

Marek Laskowski,[1] Amy L. Greer,[2] Seyed M. Moghadas[3]

[1] *Bartlett School of Graduate Studies, University College London, London, United Kingdom*

[2] *Department of Population Medicine, Ontario Veterinary College, University of Guelph, Guelph, Ontario, Canada*

[3] *Agent-Based Modelling Laboratory, York University, Toronto, Ontario, Canada*

Details of the Model structure

The basic functionality of the simulation is captured in Figure S1. The model is initialized with individual agent characteristics that are drawn from pertinent distributions (see Figures S3 – S5), and agents are assigned homes, workplaces, and school classrooms in the schedule module of the simulations. In each scenario, an infectious agent was randomly chosen to seed the simulation in the pre-symptomatic infectious state ***P***. Simulations were run and agent disease states updated in increments of 1 hour as the simulated unit of time in simulations. For more details, the reader may consult [1].

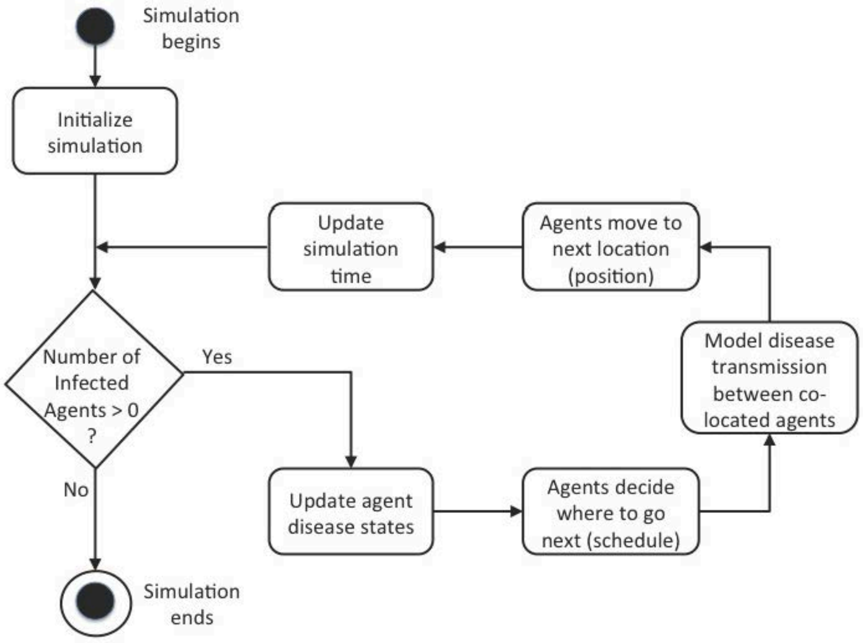

Figure S1. Basic simulation flow.

Agent schedules define relationships between agents and positions in the lattice environment as a function of time, permitting the probabilistic spread of disease between co-located agents. Positions include homes, school/classrooms, workplaces, and public arena. The schedules dictate agent relationships (changing in time) with particular positions or locations in the lattice, and include random movements that resemble a Levy-flight, with the probability of being present at a more distant location on the next time step decreasing exponentially [1].

Disease Model States and Parameters

For reference, the states used in the compartmental disease model are summarized in Table S1. The values of model parameters, their meaning, and their respective justification and ranges are summarized in Table S2.

Table S1. Summary of disease model states and time-spent in each state.

State variable	Description	Next State
$\boldsymbol{S}$	Susceptible	$\boldsymbol{E}$ if exposed or $\boldsymbol{S_P}$ if identified as a contact
$\boldsymbol{S_P}$	Susceptible, receiving prophylaxis	$\boldsymbol{S}$ if prophylaxis course ends or $\boldsymbol{E_P}$ if exposed
$\boldsymbol{E}$	Exposed to infection	$\boldsymbol{P}$ if exposed period ends or $\boldsymbol{E_P}$ if identified as a contact
$\boldsymbol{E_P}$	Exposed to infection, receiving prophylaxis	$\boldsymbol{E_X}$ if prophylaxis course ends or $\boldsymbol{P_P}$ if exposed period ends
$\boldsymbol{E_X}$	Exposed to infection, previously received prophylaxis	$\boldsymbol{P_X}$ if exposed period ends or $\boldsymbol{E_P}$ if identified as a contact
$\boldsymbol{P}$	Pre-symptomatic infection	$\boldsymbol{I_U}$ if pre-symptomatic period ends or $\boldsymbol{P_P}$ if identified as a contact
$\boldsymbol{P_P}$	Pre-symptomatic infection, receiving prophylaxis	$\boldsymbol{I_P}$ or $\boldsymbol{I_{PT}}$ if pre-symptomatic period ends or $\boldsymbol{P_X}$ if prophylaxis course ends
$\boldsymbol{P_X}$	Pre-symptomatic infection, previously received prophylaxis	$\boldsymbol{I_X}$ or $\boldsymbol{I_{XT}}$ if pre-symptomatic period ends or $\boldsymbol{P_P}$ if identified as a contact
$\boldsymbol{I_U}$	Infectious, untreated	$\boldsymbol{I_T}$ if seeks treatment or identified as a contact or $\boldsymbol{R_U}$ if infectious period ends
$\boldsymbol{I_T}$	Infectious, treated	$\boldsymbol{R_T}$
$\boldsymbol{I_P}$	Infectious, receiving prophylaxis	$\boldsymbol{I_{PT}}$ if identified as a contact or $\boldsymbol{R_U}$ if infectious period ends
$\boldsymbol{I_{PT}}$	Infectious, received prophylaxis	$\boldsymbol{R_T}$

	then treatment	
$\boldsymbol{I_X}$	Infectious, previously received prophylaxis	$\boldsymbol{I_{XT}}$ if identified as a contact or $\boldsymbol{R_U}$ if infectious period ends
$\boldsymbol{I_{XU}}$	Infectious, previously received prophylaxis, untreated	$\boldsymbol{I_{XT}}$ if seeks treatment or identified as a contact, or $\boldsymbol{R_U}$ if infectious period ends
$\boldsymbol{I_{XT}}$	Infectious, previously received prophylaxis, treated	$\boldsymbol{R_T}$
$\boldsymbol{R_U}$	Recovered, untreated (unidentified)	None
$\boldsymbol{R_T}$	Recovered, treated (identified)	None

If the agent is not receiving prophylaxis at the time that the pre-symptomatic period ends (i.e., the agent is in the $\boldsymbol{P}$ state), the agent will transition to the $\boldsymbol{I_U}$ state. It is assumed that agents receiving prophylaxis at the time that the pre-symptomatic period ends (i.e., the agent is in the $\boldsymbol{P_P}$ state) will either develop symptoms and begin treatment immediately (as they are still under medical care), or have significantly milder symptoms [15] and will not seek treatment. If the agent was previously receiving prophylaxis at the time that the pre-symptomatic period ends (i.e. the agent is in the $\boldsymbol{P_X}$ state), then the agent will transition to a stage that symptoms are mild and will therefore not seek further treatment ($\boldsymbol{I_{XU}}$) or will transition to a symptomatic stage that may seek treatment.

Table S2. Description of model parameters and their values.

**Parameter Description**	**Value(s)**	**Notes**	**Reference, assumption**
Infection transmission probability (per hour)	Tuned such that $R_0 = 1.6$ $R_0 = 2.2$ $R_0 = 2.8$	Base probability of infection transmission between one fully susceptible and one fully infectious individual if they are in contact for one hour	[5, 6]
Mean infectious period (days)	3.38	Infectious period is sampled from a log-normal distribution	[7]
Mean exposed period (days)	1.5	Exposed period is sampled from a uniform distribution	[8]
Mean pre-symptomatic period (days)	0.5	pre-symptomatic period is sampled from a log-normal distribution	[9]
Mean delay for start of	1, 2, 3	This delay is sampled from a	varied in scenarios

treatment in identified cases (days)		uniform distribution with the given mean	
Minimum delay in seeking treatment post-symptoms (days)	0.5	This delay is sampled from a uniform distribution	assumed
Infectiousness of pre-symptomatic stage relative to symptomatic stage	0.5	Pre-symptomatic is 50% less infectious than symptomatic	assumed
Probability of a close contact receiving prophylaxis or treatment (if the contact is already infectious)	between 0 and 1	An identified infectious case seeking treatment will have its close contacts within the last 24 hours traced. Each close contact will be offered prophylaxis or treatment (if infectious) with the stated probability.	varied in scenarios
Probability of an infected agent seeking treatment	between 0 and 1	The probability that an infectious individual will seek treatment and become an identified infectious case	varied in scenarios
Number of weeks that targeted prophylaxis strategy was implemented in the model	3, 5	After the treatment of first identified infectious case, prophylaxis was offered to close contacts for 3 or 5 weeks. Treatment was offered throughout the outbreak	varied in scenarios
Probability that a previously prophylaxed (but now infectious agent) will not seek treatment	0.65	The infectious agent (previously prophylaxed) will not seek treatment	[10]
Treatment duration (days)	The same as infectious period as sampled from the log-normal distribution	Entire infectious period for each individual	assumed
Prophylaxis duration (days)	7	Assumed less than 10 days on average to account for possible drop-out	assumed
Time-lag between two subsequent courses of prophylaxis for a close contact (days)	1	A second course of prophylaxis will require a minimum 1 day time lag from the previous course	assumed
Window of opportunity for effective treatment (days)	2	Treatment will not be offered to close contacts identified beyond their first 2 days following symptoms onset. They can receive	assumed

		treatment if they seek treatment.	
Reduction of susceptibility due to prophylaxis	0.3	Reduction in probability of contracting the disease	[13, 14]
Infectiousness reduction for infected agents receiving prophylaxis	0.6	Reduction in probability of transmitting the disease	[13, 14]
Infectiousness reduction for infected agents receiving treatment only	0.6	reduction in probability of transmitting the disease since the start of treatment	[10]
Infectiousness reduction for infected agents receiving treatment following prophylaxis	0.84	Reduction in probability of transmitting the disease	1 – (infectiousness with prophylaxis) multiplied by (infectiousness with treatment) [5, 14]

Infectious period distribution

The values of exposed and infectious periods were taken from estimated ranges in the published literature specific to the 2009 pandemic, with an exposed period of 1-2 days [16]. We sampled the duration of infectiousness from a log-normal distribution (shown in Figure S2) with the mean of 3.38 days [7].

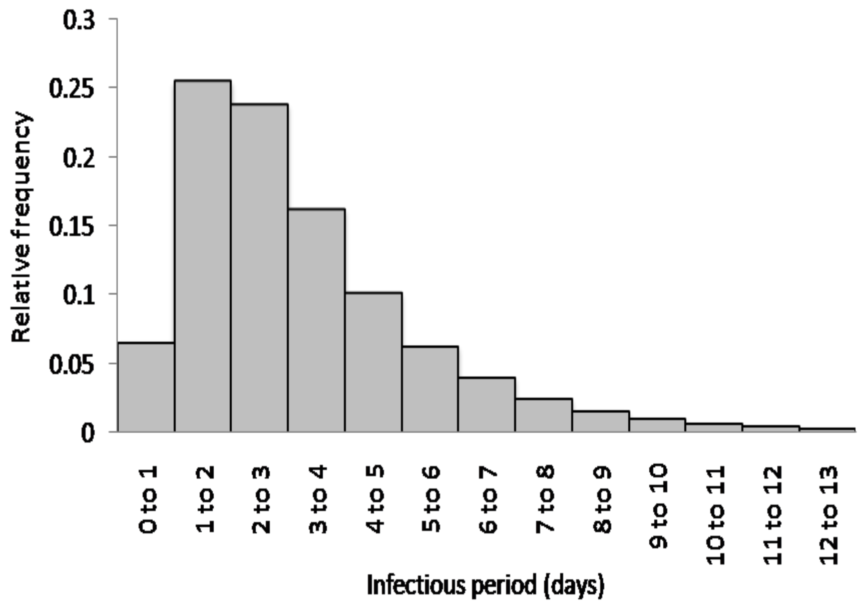

Figure S2. Relative frequency of infectious periods over 100,000 samples.

Demographic distributions

The agent demographics and household compositions used in both RC [2] and SD [3] scenarios are summarized in Figures S3-S5.

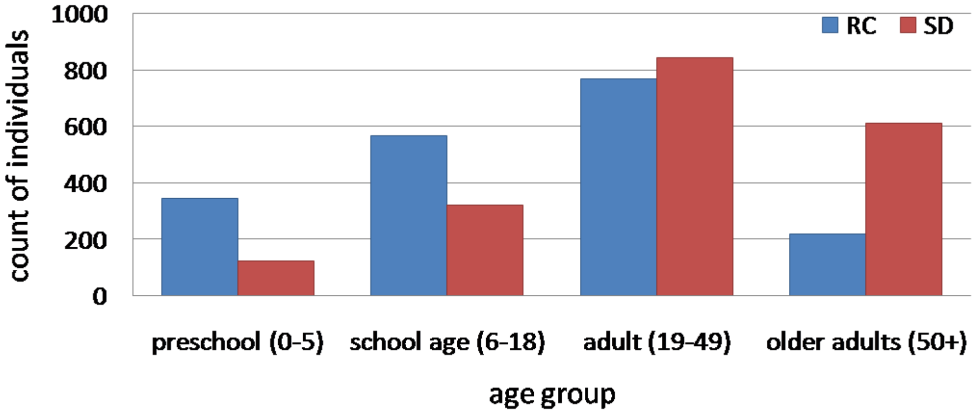

Figure S3. Comparison of age group sizes between RC and SD scenarios.

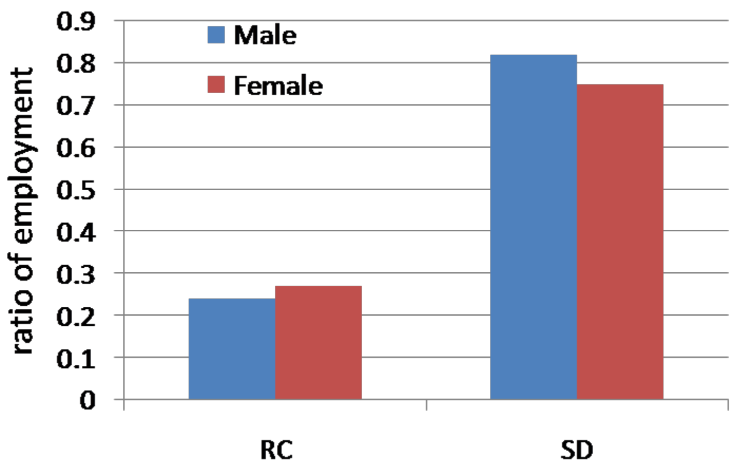

Figure S4. Comparison of ratio of employed males and females to all working-age (18-64) in both RC and SD scenarios.

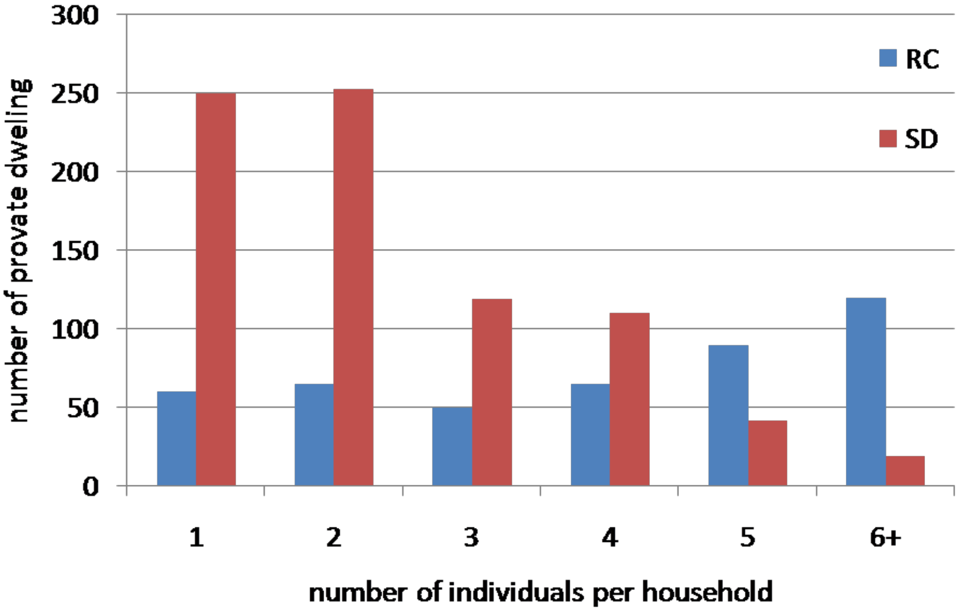

Figure S5. Comparison of household sizes between RC and SD scenarios.

From Figure S5 one can see that the urban shifted demographics (SD) scenario had more total households than the remote community (RC) scenario to accommodate the same population size. This is a result of the SD having a lower mean household size, which is characteristic of the household demographics in Winnipeg compared to the demographics in RC [4]. In addition to households, there are 12 classrooms and 129 workplaces in both RC and SD scenarios [1].

Delay in Start of Treatment

We obtained the epidemiological data for laboratory confirmed cases of the 2009 H1N1 influenza pandemic virus in the province of Manitoba, Canada. A laboratory confirmed case was an individual with influenza-like symptoms or severe respiratory illness who tested positive for H1N1 influenza A virus by viral culture or by real-time reverse-transcriptase PCR (RT-PCR). For each confirmed case, the identification date was recorded as the earliest of the dates associated with the onset of symptoms, specimen collection, hospital admission, and ICU admission. Data included dates associated with the initiation of critical care and antiviral use. Figure S6 illustrates the delay in start of treatment post symptoms onset for treated cases of H1N1 in northern Manitoba, where many of remote and isolated communities in the province are located. The mean of delay in start of treatment post symptoms is 3.5 days. Data use was approved by the Human Research Ethics Board of the University of Manitoba (H2009:339), and Health Information Privacy Committee of Manitoba (2009/2010-40), Canada.

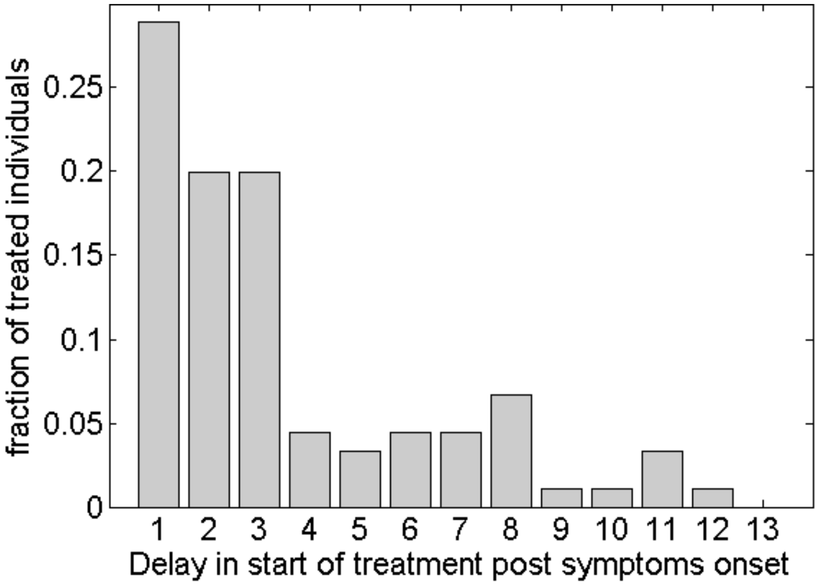

Figure S6. Fraction of H1N1 infection treated with antiviral drugs in northern Manitoba with delay in start of treatment.

Summary of Results

For evaluating antiviral strategies, we considered three scenarios for treatment of identified infectious cases, corresponding to one day, two days, and three days delay for the initiation of treatment after the onset of symptoms. For each identified infectious case, the delay in start of treatment was sampled from a uniform distribution with a mean corresponding to the delay in the simulated scenarios. The scenarios for longer delay in start of treatment are closer to the average of delay in epidemiological data reported for the 2009 H1N1 pandemic in the northern Manitoba region, Canada. These scenarios were simulated to project the relative, and cumulative age-specific attack rates in the absence of prophylaxis for both the RC and SD models (Figures 1-2 in the main text). For each scenario, we then implemented post-exposure prophylaxis of close contact for three weeks and five weeks after the first infectious case was identified for treatment. No prophylaxis was offered beyond the end of term (in both three and five weeks scenarios), but those who started a course of prophylaxis towards the end of program completed their antiviral regimen. The results for three weeks are shown in figures 3-5 in the main body. The resulting attack rates and wasteful use of prophylaxis for five weeks prophylaxis strategy are presented in Figures S7 and S8 for the RC and SD models, respectively. Effective drug usage for five weeks scenario (i.e., treatment and prophylaxis courses combined) for both RC and SD are shown in Figure S9. Figure S10 shows the number of drugs used as prophylaxis for previously infected individuals (considered as wasteful use of drugs) with varying levels of treatment for this strategy in both RC and SD models.

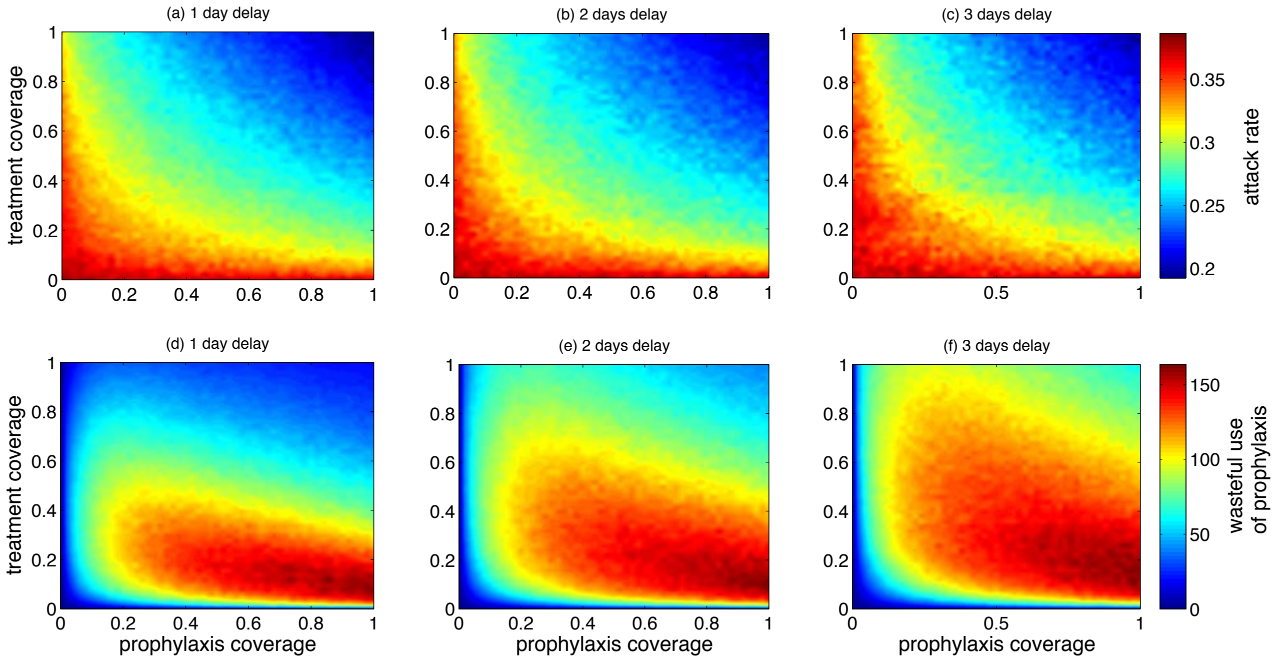

Figure S7. The projected effect of combining antiviral treatment of identified infectiouis cases with prophylaxis of close contacts on population attack rates (a-c) and wasteful use of drugs (d-f) in the RC demographics when antiviral treatment delays (for identified infectious cases) range from 1 day to 3 days post symptom onset. Duration of prophylaxis strategy: 5 weeks.

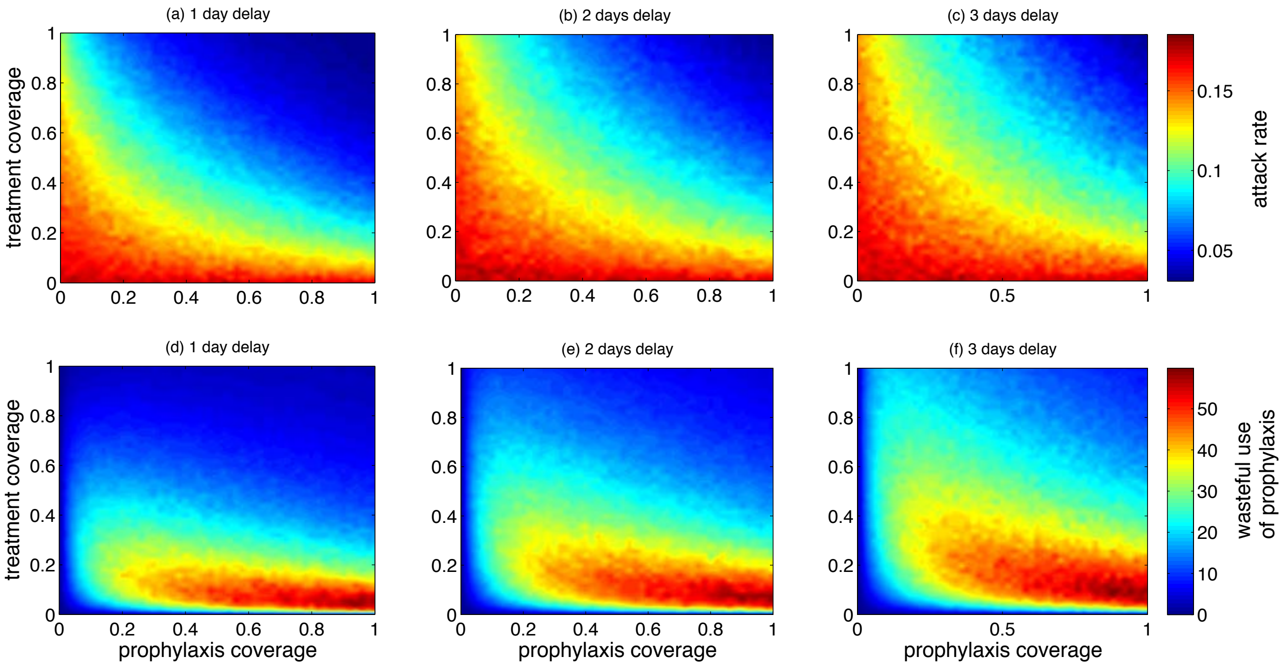

Figure S8. The projected effect of combining antiviral treatment of identified infectious cases with prophylaxis of close contacts on population attack rates (a-c) and wasteful use of drugs (d-f) in the

SD demographics when antiviral treatment delays (for identified infectious cases) range from 1 day to 3 days post symptom onset. Duration of prophylaxis strategy: 5 weeks.

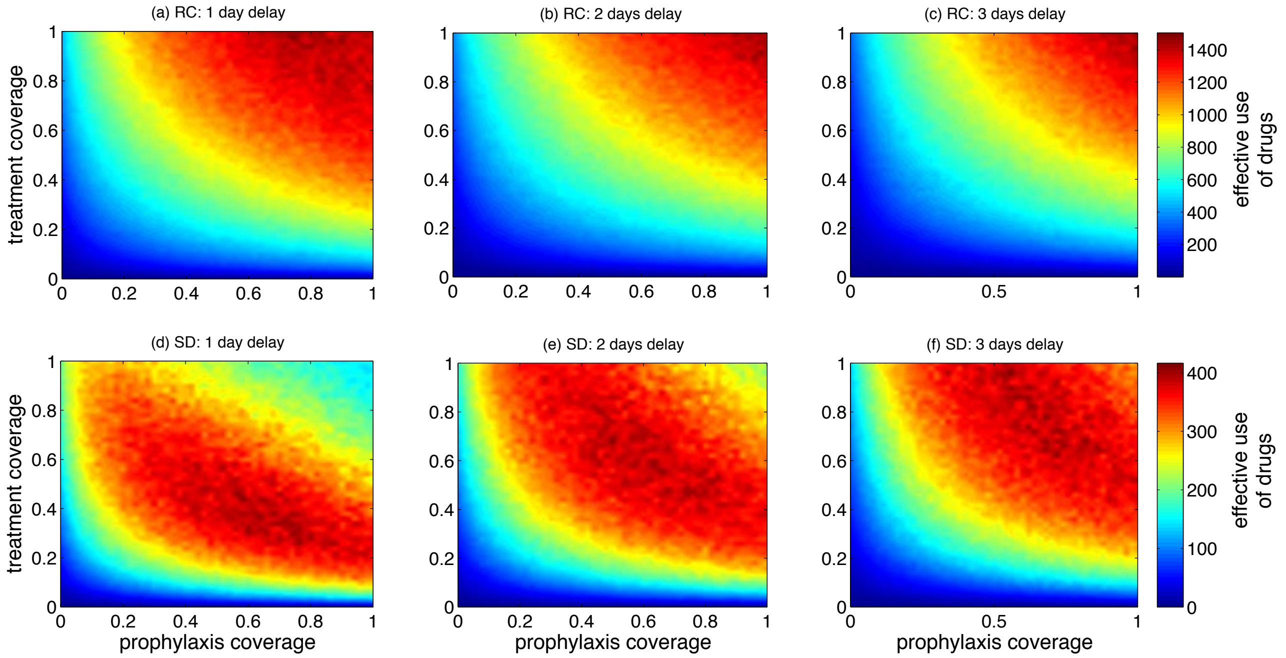

Figure S9. The projected effective drug usage when combining antiviral treatment of identified infectious cases (y-axis) with prophylaxis of close contacts (x-axis) in the remote community model (RC) (a-c) and the shifted demographics model (SD) (d-f). Antiviral treatment delays (for identified infectious cases) range from 1 day to 3 days post symptom onset. Duration of prophylaxis strategy: 5 weeks.

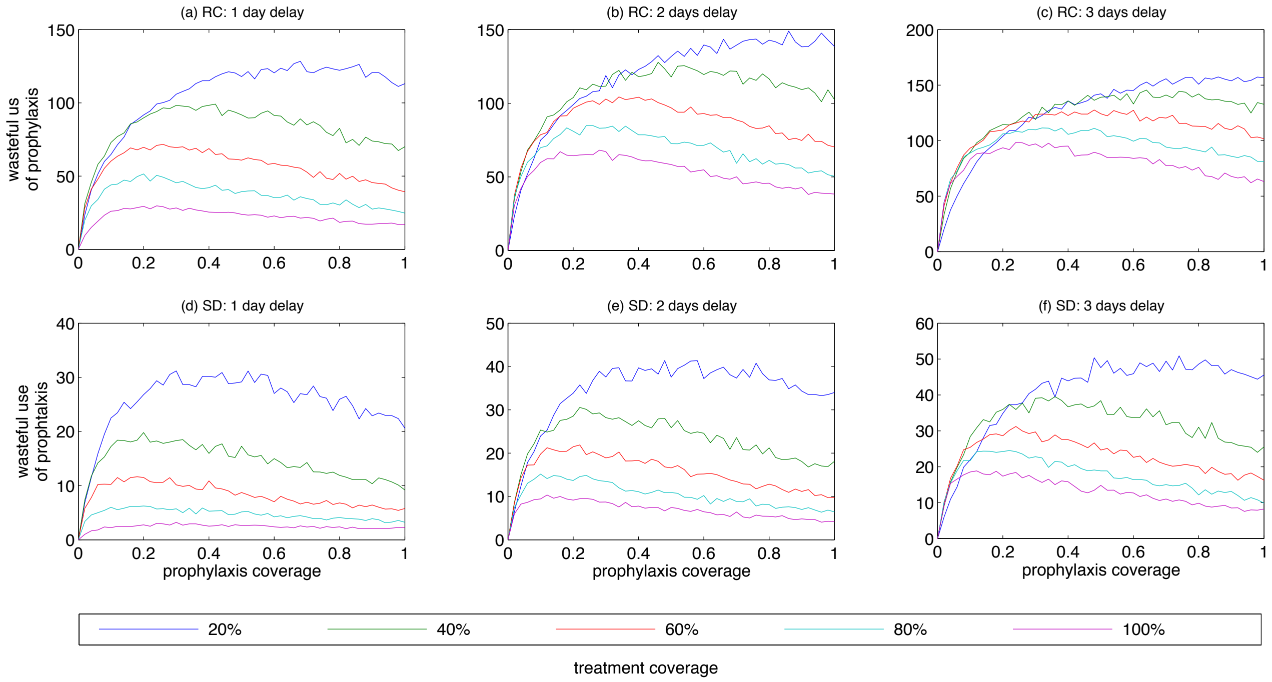

Figure S10. The projected wasteful use of prophylaxis for different treatment levels in the RC (a-c) and SD (d-f) demographics when antiviral treatment delays (for identified infectious cases) range from 1 day to 3 days post symptom onset. Duration of prophylaxis strategy: 5 weeks.

<u>Prophylaxis use per identified infectious case</u>

We analyzed independent realizations to determine the average number of prophylaxis used per infectious case in simulated scenarios. Figures S11 and S12 presents the average number of prophylaxis courses used for close contacts of an identified infectious case in RC and SD scenarios for 3 weeks (Fig. S11) and 5 weeks (Fig. S12) prophylaxis strategy.

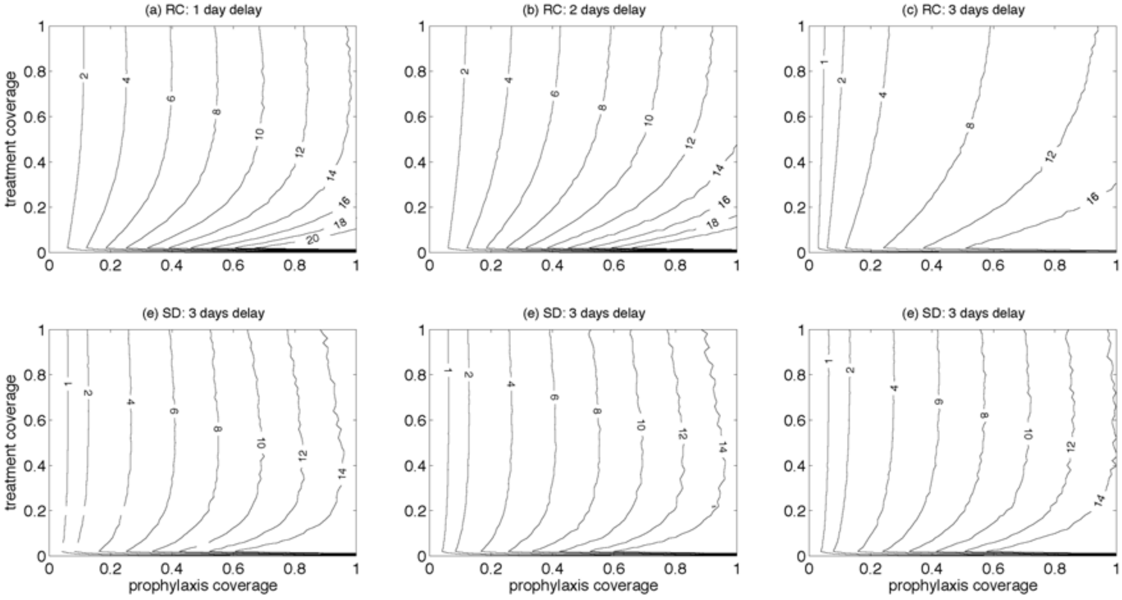

Figure S11. Average prophylaxis courses given per identified infectious case in RC scenarios (a-c), and SD scenarios (d-f). Duration of prophylaxis: 3 weeks.

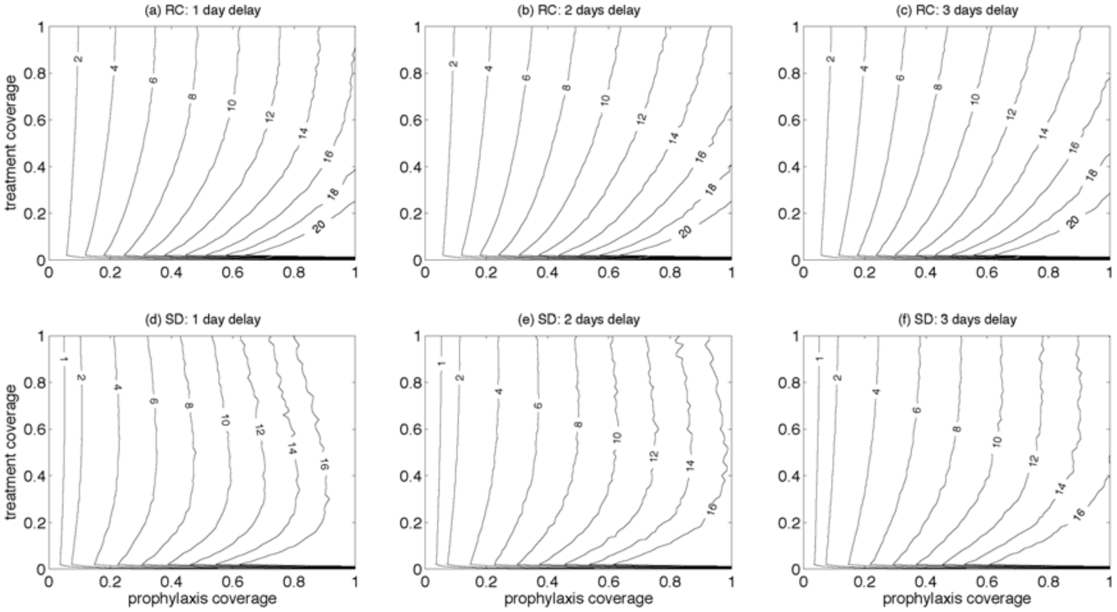

Figure S12. Average prophylaxis courses given per identified infectious case in RC scenarios (a-c), and SD scenarios (d-f). Duration of prophylaxis: 5 weeks.

Variations in the reproduction number

We tested the effect of changing the reproduction number on the model outcomes for antiviral and prophylaxis strategies for 3 and 5 weeks scenarios. Qualitatively, the simulation results are consistent with the baseline reproduction number $R_0 = 2.2$ described in the main text. These simulations are presented in the following sections for two different reproduction numbers.

Simulations for $\boldsymbol{R_0 = 1.6}$

The results of these simulations are shown in Figures S13 – S16; all the assumptions and other parameter values/ranges are the same as those used for the baseline scenarios of $\boldsymbol{R_0 = 2.2}$.

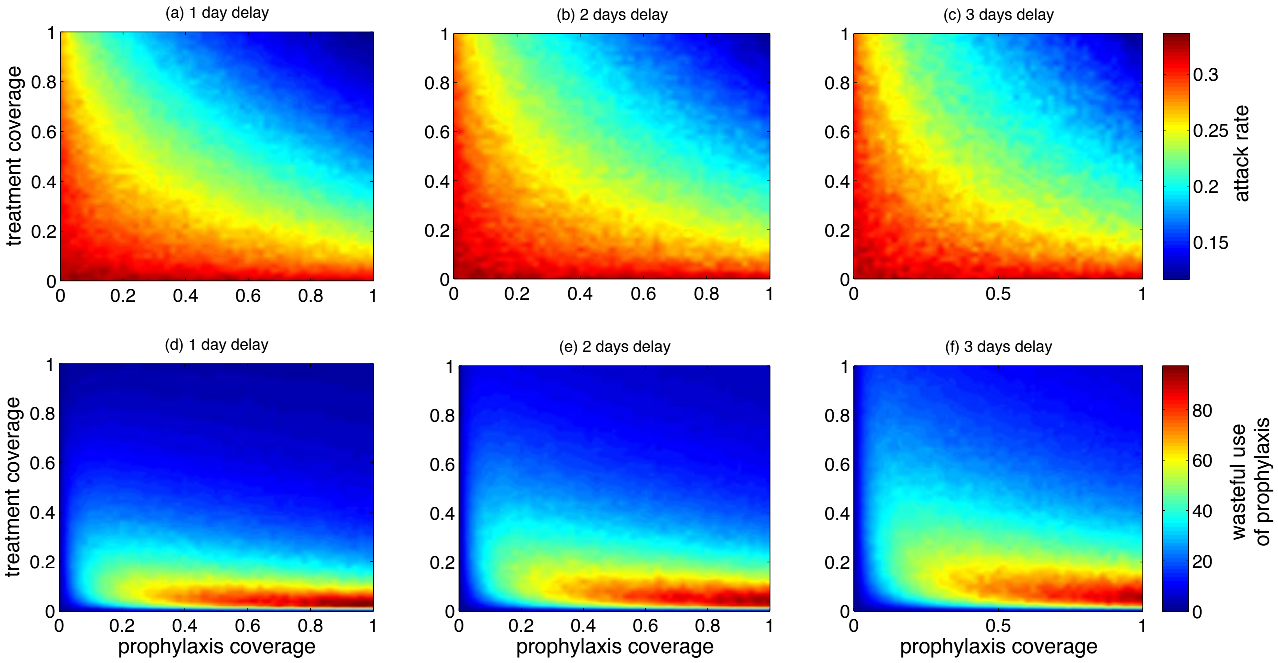

Figure S13. The projected effect of combining antiviral treatment of identified infectious cases with prophylaxis of close contacts on population attack rates (a-c) and wasteful use of drugs (d-f) in the RC demographics when antiviral treatment delays (for identified infectious cases) range from 1 day to 3 days post symptom onset. Duration of prophylaxis strategy: 3 weeks.

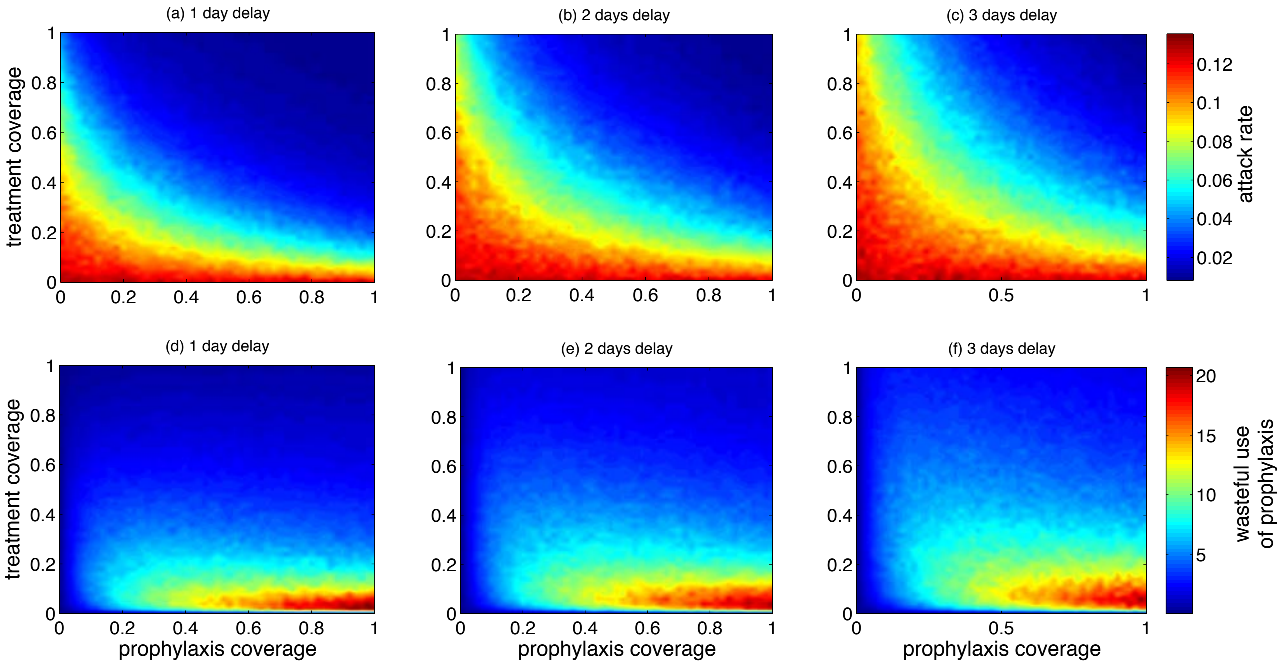

Figure S14. The projected effect of combining antiviral treatment of identified infectious cases with prophylaxis of close contacts on population attack rates (a-c) and wasteful use of drugs (d-f) in the SD demographics when antiviral treatment delays (for identified infectious cases) range from 1 day to 3 days post symptom onset. Duration of prophylaxis strategy: 3 weeks.

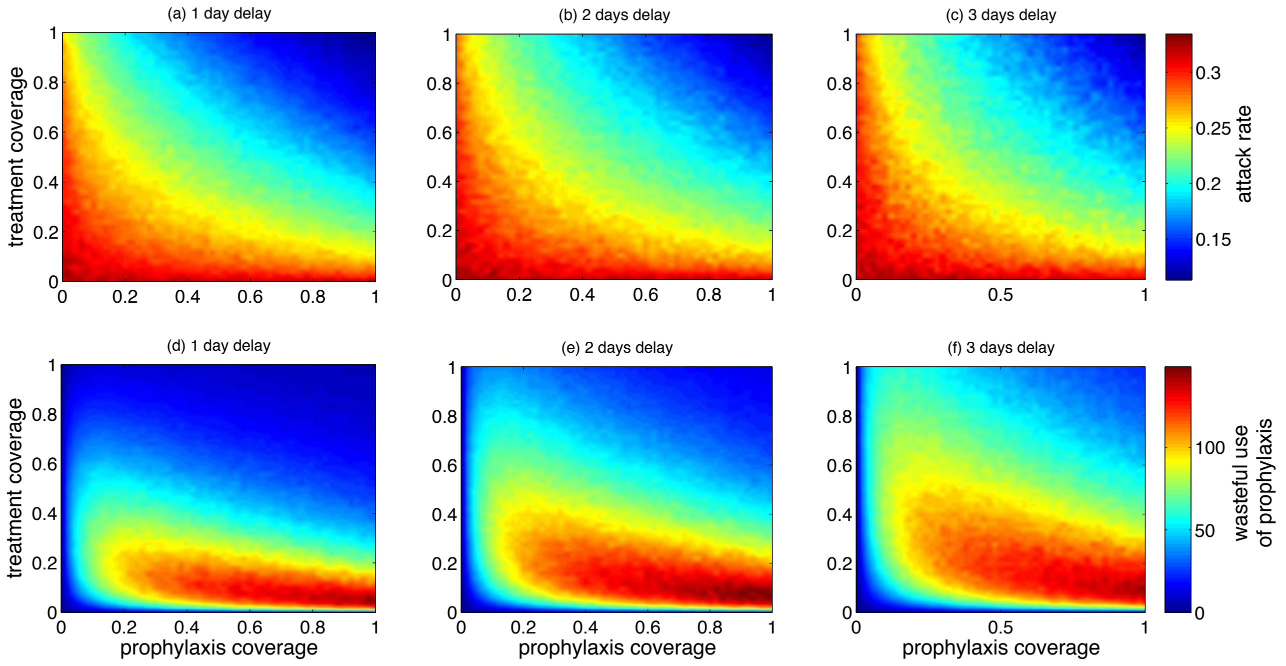

Figure S15. The projected effect of combining antiviral treatment of identified infectious cases with prophylaxis of close contacts on population attack rates (a-c) and wasteful use of drugs (d-f) in the RC demographics when antiviral treatment delays (for identified infectious cases) range from 1 day to 3 days post symptom onset. Duration of prophylaxis strategy: 5 weeks.

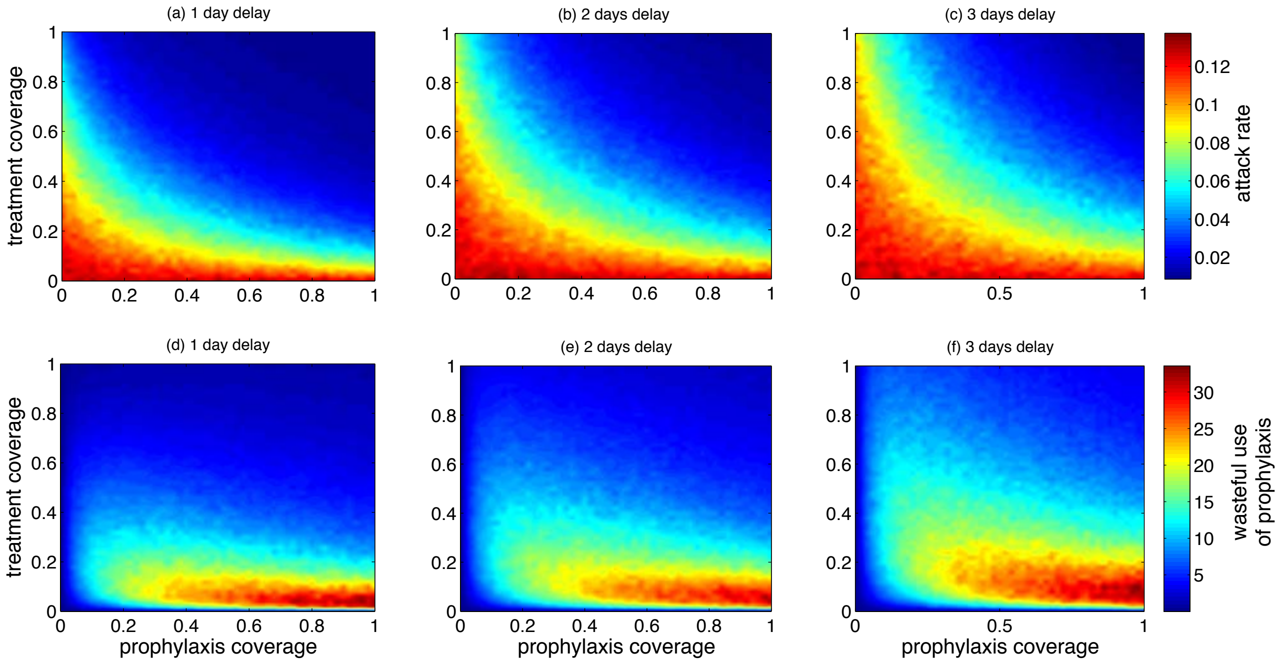

Figure S16. The projected effect of combining antiviral treatment of identified infectious cases with prophylaxis of close contacts on population attack rates (a-c) and wasteful use of drugs (d-f) in the SD demographics when antiviral treatment delays (for identified infectious cases) range from 1 day to 3 days post symptom onset. Duration of prophylaxis strategy: 5 weeks.

Simulations for $R_0 = 2.8$

The results of these simulations are shown in Figures S17 - S20; all the assumptions and other parameter values/ranges are the same as those used for the baseline scenarios of $R_0 = 2.2$.

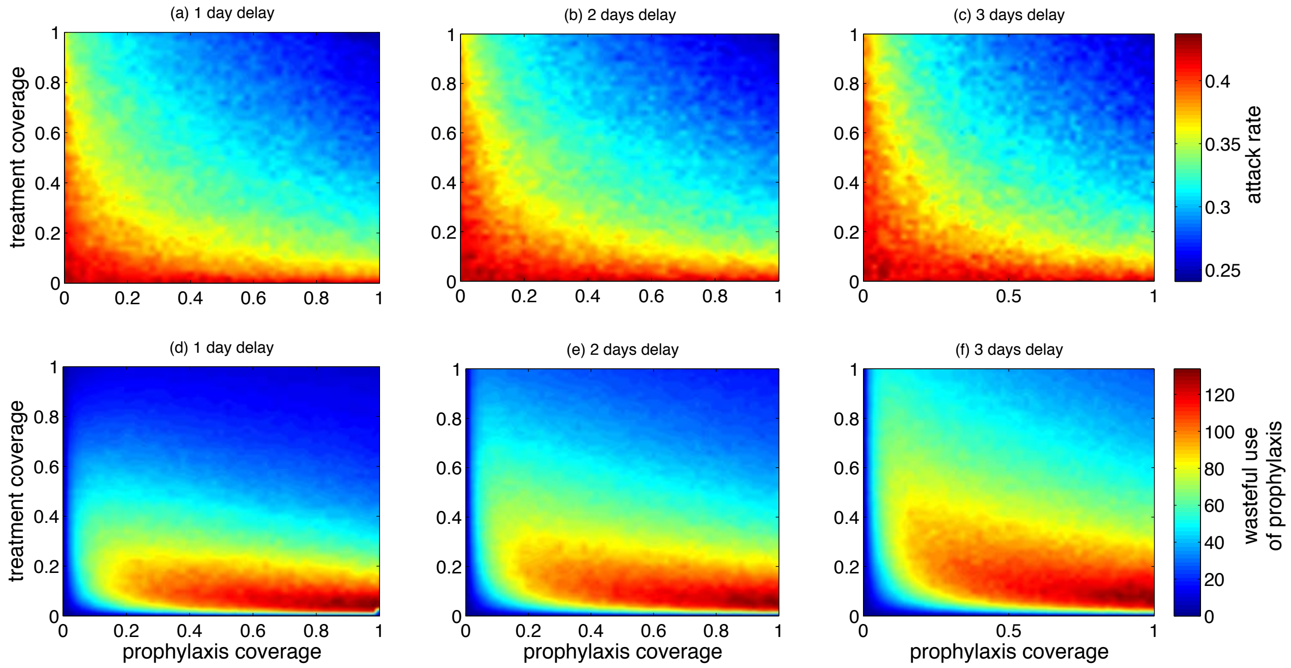

Figure S17. The projected effect of combining antiviral treatment of identified infectious cases with prophylaxis of close contacts on population attack rates (a-c) and wasteful use of drugs (d-f) in the RC demographics when antiviral treatment delays (for identified infectious cases) range from 1 day to 3 days post symptom onset. Duration of prophylaxis strategy: 3 weeks.

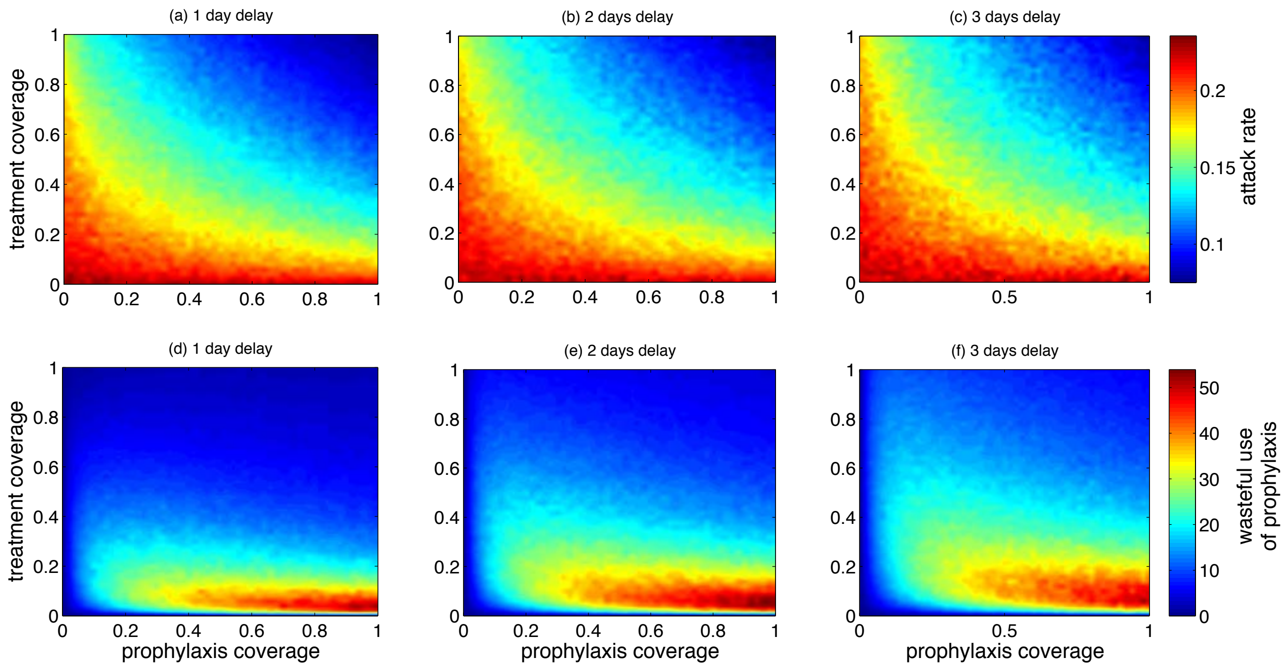

Figure S18. The projected effect of combining antiviral treatment of identified infectious cases with prophylaxis of close contacts on population attack rates (a-c) and wasteful use of drugs (d-f) in the SD demographics when antiviral treatment delays (for identified infectious cases) range from 1 day to 3 days post symptom onset. Duration of prophylaxis strategy: 3 weeks.

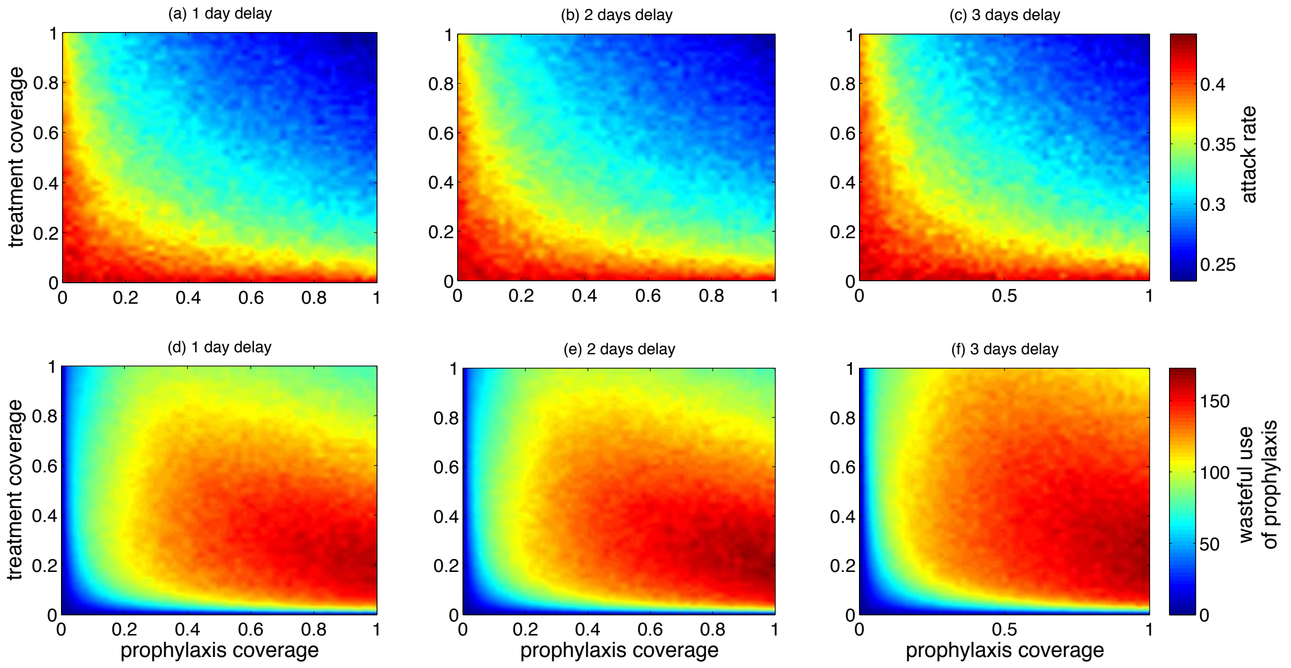

Figure S19. The projected effect of combining antiviral treatment of identified infectious cases with prophylaxis of close contacts on population attack rates (a-c) and wasteful use of drugs (d-f) in the RC demographics when antiviral treatment delays (for identified infectious cases) range from 1 day to 3 days post symptom onset. Duration of prophylaxis strategy: 5 weeks.

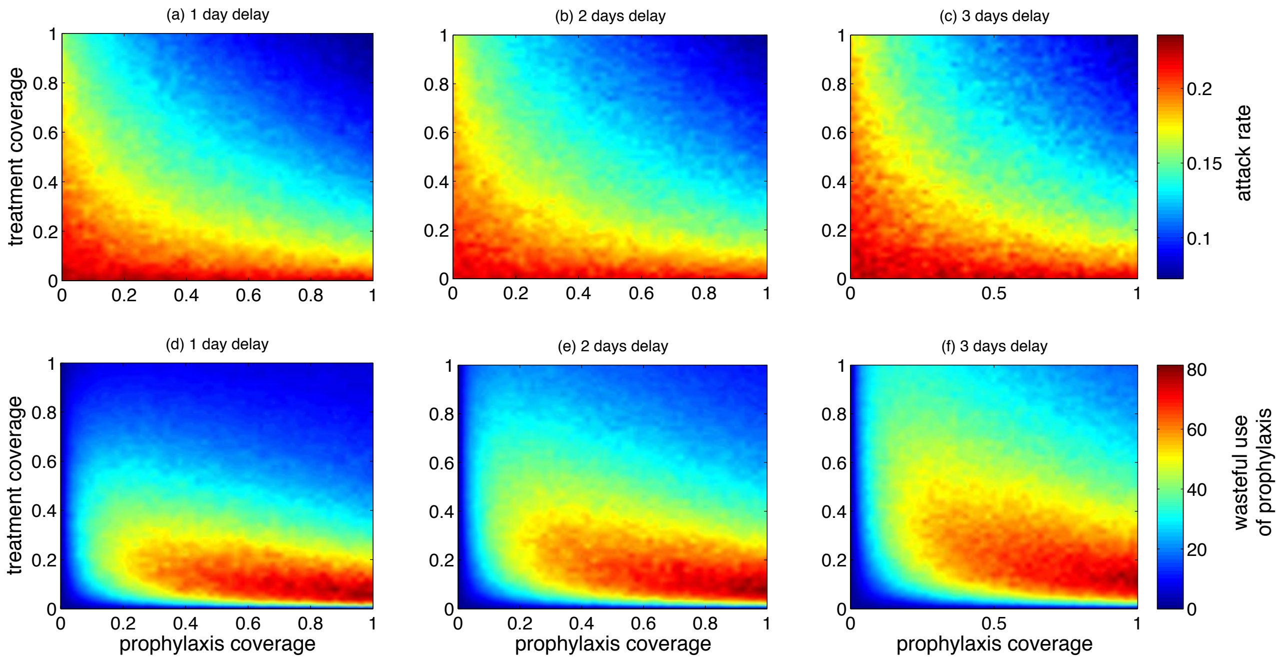

Figure S20. The projected effect of combining antiviral treatment of identified infectious cases with prophylaxis of close contacts on population attack rates (a-c) and wasteful use of drugs (d-f) in the SD demographics when antiviral treatment delays (for identified infectious cases) range from 1 day to 3 days post symptom onset. Duration of prophylaxis strategy: 5 weeks.

References

1. Laskowski M, Mostaço-Guidolin LC, Greer AL, et al. The impact of demographic variables on disease spread: influenza in remote communities. Sci Rep 2011; 1:105.

2. Statistics Canada. 2007. Manitoba (Code4622048) (table). 2006 Community Profiles. 2006 Census. Statistics Canada Catalogue no. 92-591-XWE. Ottawa. Released March 13, 2007.

3. Statistics Canada. 2007. Winnipeg, Manitoba (Code4611040) (table). 2006 Community Profiles. 2006 Census. Statistics Canada Catalogue no. 92-591-XWE. Ottawa. Released March 13, 2007.

4. Number of Rooms (12) and Household Size (9) for Occupied Private Dwellings of Canada, Provinces, Territories, Census Divisions and Census Subdivisions, 2006 Census - 20% Sample

Data. Statistics Canada, 2006 Census of Population, Statistics Canada catalogue no. 97-554-XCB2006016 (IRI Code4622048).

5. Mostaco-Guidolin LC, Bowman CS, Greer AL, Fisman DN, Moghadas SM, Transmissibility of the 2009 H1N1 pandemic in remote and isolated Canadian communities: a modelling study, British Medical Journal - Open, 2: e001614.
6. Mostaco-Guidolin LC, Greer AL, Sander B, Wu J, Moghadas SM, Variability in transmissibility of the 2009 H1N1 pandemic in Canadian communities. BMC Research Notes 4: 537.
7. Tuite, A. R., et al., Estimated epidemiologic parameters and morbidity associated with pandemic H1N1 influenza. CMAJ 2010; 182: 131–136.
8. Lessler, J., et al., Incubation periods of acute respiratory viral infections: a systematic review. Lancet Infect. Dis. 2009; 9: 291–300.
9. Gu Y, Komiya N, Kamiya H, Yasui Y, Taniguchi K, Okabe N, Pandemic (H1N1) 2009 transmission during presymptomatic phase, Japan, Emerg Infect Dis. 2011; 17: 1737-1739.
10. Ferguson NM, et al., Strategies for containing an emerging influenza pandemic in Southeast Asia. Nature 2005; 437: 209-214.
11. Reed C, Angulo FJ, Swerdlow DL, Lipsitch M, Meltzer MI, Jernigan D, Finelli L, Estimates of the prevalence of pandemic (H1N1) 2009, United States, April–July 2009, Emerg Infect Dise 2009; 15: 2004-2007.
12. Gojovic MZ, Sander B, Fisman D, Krahn MD, Bauch CT, Modelling mitigation strategies for pandemic (H1N1) 2009, CMAJ 2009, 181: 673-680.
13. Halloran ME, Hayden FG, Yang Y, Longini IM Jr, Monto AS, Antiviral effects on influenza viral transmission and pathogenicity: observations from household-based trials, Am J Epidemiol 2007; 165(2): 212-221
14. Yang, Y., et al., Design and evaluation of prophylactic interventions using infectious disease incidence data from close contact groups. Journal of the Royal Statistical Society 2006; 55: 317–330.
15. Longini, I. M. Jr, Halloran, M. E., Nizam, A. & Yang, Y. Containing pandemic influenza with antiviral agents. Am. J. Epidemiol 2004; 159: 623-633.

16. Lessler J, Reich NG, Cummings DAT, et al. Outbreak of 2009 pandemic influenza A (H1N1) at a New York City school. N Engl J Med 2009; 361: 2628-2636.

17. Moghadas SM, Day T, Bauch C, et al. Modelling of pandemic influenza: a guide for the perplexed, CMAJ 2009; 181: 171-173.

18. Svensson Å, A note on generation times in epidemic models. Math Biosci 2007; 208: 300-311.

19. Donnelly CA, Finelli L,, Cauchemez S, et al. Serial intervals and the temporal distribution of secondary infections within households of 2009 Pandemic Influenza A (H1N1): implications for influenza control recommendations. Clin Infect Dis 2010; 52: S123-130.
